# The Prospective Use of Brazilian Marine Macroalgae in Schistosomiasis Control

**DOI:** 10.3390/md19050234

**Published:** 2021-04-22

**Authors:** Erika M. Stein, Sara G. Tajú, Patrícia A. Miyasato, Rafaela P. de Freitas, Lenita de F. Tallarico, Guilherme S. dos Santos, Giovana L. F. Luiz, Henrique K. Rofatto, Fábio N. V. da Silva, Pio Colepicolo, Arthur L. Macedo, Carlos A. Carollo, Eliana Nakano

**Affiliations:** 1 Laboratory of Biochemistry and Molecular Biology of Marine Algae—Chemistry Institute, São Paulo University, São Paulo 05508-090, SP, Brazil; stein.erika.m@gmail.com (E.M.S.); piocolep@iq.usp.br (P.C.); 2 Laboratory of Parasitology, Butantan Institute, São Paulo 05503-900, SP, Brazil; sarabibitaju@gmail.com (S.G.T.); patricia.aoki@butantan.gov.br (P.A.M.); rafaela.freitas@butantan.gov.br (R.P.d.F.); lenita.tallarico@butantan.gov.br (L.d.F.T.); gsennasantos@gmail.com (G.S.d.S.); giovana.luiz@butantan.gov.br (G.L.F.L.); hkrofatto@hotmail.com (H.K.R.); vienufabio@gmail.com (F.N.V.d.S.); 3 Laboratory of Natural Products and Mass Spectrometry—LaPNEM, Faculty of Pharmaceutical Sciences, Food and Nutrition, Federal University of Mato Grosso do Sul, Campo Grande 79070-900, MS, Brazil; arthur.ladeira@ufms.br (A.L.M.); carlos.carollo@ufms.br (C.A.C.)

**Keywords:** *Laurencia*, *Laurenciella*, *Dictyota*, sesquiterpenes, diterpenes, metabolomic analysis, GC–MS, Pattern Hunter test, Pearson correlation test

## Abstract

Schistosomiasis is a parasitic disease that affects more than 250 million people. The treatment is limited to praziquantel and the control of the intermediate host with the highly toxic molluscicidal niclosamide. Marine algae are a poorly explored and promising alternative that can provide lead compounds, and the use of multivariate analysis could contribute to quicker discovery. As part of our search for new natural compounds with which to control schistosomiasis, we screened 45 crude extracts obtained from 37 Brazilian seaweed species for their molluscicidal activity against *Biomphalaria glabrata* embryos and schistosomicidal activities against *Schistosoma mansoni*. Two sets of extracts were taxonomically grouped for metabolomic analysis. The extracts were analyzed by GC–MS, and the data were subjected to Pattern Hunter and Pearson correlation tests. Overall, 22 species (60%) showed activity in at least one of the two models. Multivariate analysis pointed towards 3 hits against *B. glabrata* veliger embryos in the *Laurencia/Laurenciella* set, 5 hits against *B. glabrata* blastula embryos, and 31 against *S. mansoni* in the Ochrophyta set. Preliminary annotations suggested some compounds such as triquinane alcohols, prenylated guaianes, dichotomanes, and xenianes. Despite the putative identification, this work presents potential candidates and can guide future isolation and identification.

## 1. Introduction

Schistosomiasis is a parasitic disease caused by trematode worms of the *Schistosoma* genus. Endemic in 78 countries of Africa, the eastern Mediterranean, Southeast Asia, and South America, schistosomiasis affects more than 250 million people, especially in poor communities, with no access to treated water and basic sanitation [1]. These figures show that the elimination of schistosomiasis is far from being achieved.

Praziquantel is the single option available for control programs and schistosomiasis treatment. Despite its safety, praziquantel is not active against young worms and does not prevent reinfection. Resistance has not been reported yet, but the occurrence of less susceptible parasites in laboratory conditions has been observed [2]. Moreover, niclosamide is the only molluscicide used for host control; despite the effectiveness against adult mollusks and eggs, it is highly toxic to non-target species [3]. 

Natural products can be an alternative, providing leading compounds for the development of new drugs. Most bioprospection studies are focused on compounds isolated from vegetal species [4]; nevertheless, marine species can be pointed out as an alternative source of active compounds. For example, halogenated secondary metabolites, while rare in terrestrial plants, are common in marine organisms due to the abundance of chloride and bromide ions in seawater [5] and showed biological activity to different pathogens [6]. Therefore, marine macroalgae are an important source of new active compounds, especially in Brazil, where numerous species are found on the extensive coastline [7]. 

The antiparasitic activity of different marine macroalgae species has been identified in several bioprospection studies; nevertheless, most data come from studies with protozoa [8,9,10,11,12,13,14,15,16], and there is little information on the anthelmintic activity of marine macroalgae [6,7]. 

Despite the potential, bioprospection of natural products is a laborious and time-consuming task. Innovative strategies based on metabolic profiling are being studied and applied to accelerate the search for new active compounds [17]. Using multivariate statistical analysis and correlation analysis, researchers have applied the metabolomic approach with success for this purpose against some infectious and parasitic agents [18]. 

Recently, we detected the in vitro activity of seaweed extracts against *S. mansoni*. Three out of the 13 tested extracts induced lethal effects in the exposed worms [19]. In the present study, a more comprehensive trial was performed with the screening of 37 species of Brazilian macroalgae for schistosomicidal and molluscicidal activity searching for compounds of potential use in the treatment and control of schistosomiasis. We used successful metabolomics tools to annotate potential bioactive compounds of a group of seaweed extracts.

## 2. Results and Discussion

### 2.1. Biological Activity

#### 2.1.1. *Schistosoma* *Mansoni*

Potential anthelminthic compounds from Brazilian seaweed species were screened by testing crude extracts from 37 seaweed species collected at different times as part of a major study represented by many small projects. The analysis of the effects on viability and reproduction allowed the identification of 21 species with activity: 14 Rhodophyta, 4 Ochrophyta, and 3 Chlorophyta (Table 1), resulting from a total of 25 species of Rhodophyta, 8 Ochrophyta, and 4 Chlorophyta tested in vitro on *S. mansoni* adult worms. 

Thirty-one extracts from 25 species of Rhodophyta were tested for anthelmintic activity. Five species exhibited schistosomicide activity; 12 species affected reproduction with no effects on viability, and 7 species were inactive for *S. mansoni*.

All four species from the *Laurencia* complex tested in the present study—*L. aldingensis*, *L. catarinensis*, *L. dendroidea*, and *Laurenciella* sp.—exhibited schistosomicidal activity. A differential response was observed when comparing the results for different extracts from the same seaweed species depending on the solvent.

The *L. aldingensis* hexane extract induced 100% mortality in worms (females were more sensitive than males). The chloroform extract showed a weak schistosomicidal activity (20%), and the methanol extract was not active. Concerning the effects on the reproduction, the analysis of worm pairing showed 80% separation by hexane extract, and despite the weak schistosomicidal effect of the chloroform extract, it caused 100% separation of worm pairs. The *L. aldingensis* hexane and chloroform extracts also wholly inhibited the oviposition.

The *L. dendroidea* hexane and chloroform extracts induced 100% mortality in worms (females were slightly more sensitive). The *L. dendroidea* methanol extract was not active. All *L. dendroidea* extracts inhibited the reproduction: chloroform separated 100% of worm pairs, hexane 80%, and methanol 60%. Hexane and chloroform extracts also totally inhibited the oviposition. The *L. catarinensis* chloroform extract did not exhibit schistosomicidal activity; however, it induced 100% pair separation and completely inhibited the oviposition.

The chloroform extract of *Laurenciella* sp. was the only one with schistosomicide activity, with 100% mortality. Reproduction was inhibited by all extracts, with 100% separation of worm pairs exposed to hexane and chloroform extracts and 20% for methanol extract. Finally, oviposition was highly inhibited by the hexane and chloroform extracts.

The *L. catarinensis* chloroform extract did not exhibit schistosomicidal activity; however, it induced 100% pair separation and completely inhibited the oviposition.

*Ochtodes secundiramea* induced 100% mortality in male and female worms; females were slightly more sensitive than male worms. Reproduction was highly affected, with 100% couple separation and complete inhibition of oviposition. *Porphyra spiralis* induced 100% of mortality in males and female worms; male worms were slightly more sensitive. *P. spyralis* strongly affected the reproduction, inducing separation in 100% of worm couples and inhibiting the oviposition.

Some species affected only reproduction with or without effect on oviposition and no effect on the exposed worms’ viability. *Amphiroa fragilissima*, *Bostrychia tennela*, *Dichotomaria marginata*, *Gracilaria domingensis*, *Hypnea nigrescens*, *Jania rubens*, *Solieria filiformis*, *Spyridia aculeata*, and *Tricleocarpa cylindrica* caused the separation of couples and inhibited the oviposition at different levels. On the other hand, *Cryptonmenia crenulata*, *Cryptonmenia seminervis*, and *Vidalia obtusiloba* induced separation of couples without significantly reducing the number of eggs.

*Botryocladia occidentalis*, *Bryothamnion seaforthii*, *Ceratodictyon variabile*, *Gracilaria* cf. *intermedia*, *Palisada perforata*, *Palisada flagellifera*, and *Pterocladiella capillacea* did not show any effect in exposed worms.

Ten extracts from eight species of Ochrophyta were tested, thus leading to two schistosomicidal species, *D. mertensii* and *D. ciliolata*. *Dictyota mertensii* supercritical fluid induced 100% lethality after 48 h of exposure. Reproduction was positively affected by the extract, with 80% of pairs separation after 2 h of exposure and 100% after 24 h; oviposition was totally inhibited. For *Dictyota ciliolata*, a differential response was observed depending on the solvent. The schistosomicidal activity was observed for all the extracts: supercritical fluid exhibited the highest effect with 100% lethality, followed by hexane and chloroform; males were more sensitive than females exposed to the chloroform extract. The reproduction was enormously affected, with the separation of 100% of pairs and complete inhibition of the oviposition. 

*Canistrocarpus cervicornis* affected only the reproduction causing separation of 100% of couples and inhibiting oviposition. *Sargassum vulgare* only slightly affected the reproduction, inducing 20% couple separation with no effects on oviposition. No phenotypic effects on exposed worms were observed for *Colpomenia sinuosa*, *Padina gymnospora*, *Padina tetrastomatica*, or *Zonaria tournefortii*.

Chlorophyta anthelminthic activity was assessed by testing four extracts from four species on *S. mansoni* worms and showed a weak potential. One species showed schistosomicidal activity; two species affected only the reproduction, and one was an inactive species (Table 1). *Codium isthmocladum* induced 20% mortality in male worms; females were not affected. Reproductive effects were observed in pairing with 100% separation; oviposition was not affected. Reproduction was highly affected by *Caulerpa sertularioides* and *C. racemosa*, which caused 100% and 80% pair separation, respectively, and almost completely inhibited oviposition. *Caulerpa cupressoides* did not show any phenotypic effect on exposed worms.

Overall, the results suggest a nonpolar nature for the active compounds; this was especially evident when comparing the response to different extracts from the same seaweed species, as observed for *L. aldingensis*, *L. dendroidea*, *Laurenciella* sp., and *D. ciliolata*. Supercritical CO_2_ was used for *D. mertensii* and *D. ciliolata*, and the method was as efficient as hexane for the extraction of nonpolar compounds, showing similar activity on *S. mansoni*.

An inhibiting effect on worm reproduction was directly observed by egg counting. All active extracts completely inhibited the oviposition or strongly reduced the number of eggs. This is an important parameter in drug development for schistosomiasis since it is the main factor in the pathology. Furthermore, the effect on the reproduction was assessed by the observation of worm pairing. All active extracts induced separation of the worm couples. This is an additional endpoint to predict the drugs’ effects on the pathology of schistosomiasis.

A differential in the sensitivity of male and female worms was observed for most of the extracts, suggesting that active compounds may have different mechanisms of action for each sex. This sex-specific effect was also reported for the reference drug, praziquantel, both in vivo and in vitro [20,21]. A differential response to praziquantel between sexes was observed upon transcriptome analysis of male and female worms, suggesting that different molecular processes are involved [22].

On the basis of the published data, we found our studies to be the first in the assessment of seaweeds for antischistosomal effects. The only reports on seaweed anthelminthic activity described the effects of bisabolanes and sesquiterpenes isolated from the red seaweed *Laurencia scoparia* on the rodent nematoid *Nippostrongylus brasiliensis* [23,24]. 

#### 2.1.2. *Biomphalaria* *glabrata*

In this screening, crude extracts from 36 seaweed species (24 Rhodophyta, 8 Ochrophyta, and 4 Chlorophyta) were tested for molluscicide activity. The analysis of effects on *Biomphalaria glabrata* embryos at the blastula and veliger stage allowed for the identification of 22 species with activity: 16 Rhodophyta, 4 Ochrophyta, and 2 Chlorophyta (Table 1).

Rhodophyta seaweeds were assessed by testing 32 crude extracts from 24 species, resulting in 6 highly active species inducing 100% lethality in both embryo stages, 10 species affecting embryos only at the blastulae stage, and 8 inactive species.

The most active species were *Laurencia aldingensis*, *Laurencia catarinensis*, *Laurencia dendroidea*, *Laurenciella* sp., *Ochtodes secundiramea*, and *Spyridia aculeata*, which induced 100% mortality in embryos at both stages. *Amphiroa fragilissima*, *Botryocladia occidentalis*, *Cryptonmenia crenulata*, *Cryptonmenia seminervis*, *Dichotomaria marginata*, *Jania Rubens*, *Palisada flagellifera*, *Solieria filiformis*, *Tricleocarpa cylindrica*, and *Vidalia obtusiloba* were active only at the blastula stage. *Bostrychia tennela*, *Bryothamnion seaforthii*, *Ceratodictyon variabile*, *Gracilaria* cf. *intermedia*, *Gracilaria domingensis*, *Hypnea nigrescens*, *Palisada perforata*, and *Porphyra spiralis* were inactive for embryos at both stages.

When diverse extracts were tested for the same seaweed species, a differential response was observed depending on the solvent. For *Laurencia aldingensis*, the hexane extract was the most active, inducing 100% mortality in both embryo stages, followed by chloroform, which induced 100% of mortality in blastulae and 11% in veliger; the methanol extract killed 56.6% of the embryos at the blastulae stage and 2.4% at the veliger stage. *Laurenciella* sp. hexane extract was the most active, inducing 100% mortality in embryos at both stages, followed by the methanol extract with 100% and 70.3% and chloroform with 71.3% and 6.1% of dead embryos at the blastulae and veliger stages, respectively. For *Laurencia dendroidea*, there was no difference in the activity among different solvents; the hexane, chloroform, and methanol extracts were lethal to 100% of embryos at both stages.

Ochrophyta molluscicidal activity was assessed by testing nine extracts from eight seaweeds species. Two species were active to embryos at both the blastulae and veliger stages; two were active only in blastulae, and four were inactive.

*Dictyota ciliolata* and *D. mertensii* were active in both embryo stages; blastulae was the stage most sensitive to both seaweed species. *Dictyota mertensii* supercritical fluid was 100% active in the blastulae stage embryos and 9.8% in the veliger stage.

Two extracts of *Dictyota ciliolata* were tested, and a difference in the responses was observed. The chloroform extract was the most active, inducing 100% mortality in the embryos at the blastulae stage and 40.4% at the veliger stage. Hexane extract induced 100% and 11.1% mortality in embryos at the blastulae and veliger stages, respectively.

*Canistrocarpus cervicornis* and *Padina tetrastomatica* were 100% lethal to embryos at the blastulae stage and inactive at the veliger stage.

*Colpomenia sinuosa*, *Padina gymnospora*, *Sargassum vulgare*, and *Zonaria tournefortii* did not induce any effects on *B. glabrata* embryos.

Chlorophyta molluscicidal activity was assessed by testing four extracts from four species, identifying two active species. *Caulerpa racemosa* and *Codium isthmocladum* extracts showed 100% blastulae activity and no effect in the veliger stage. *Caulerpa cupressoides* and *Caulerpa sertularioides* extracts were inactive for both embryo stages.

Several seaweed species were assessed for molluscicidal activity on *B. glabrata* and *B. alexandrina* snails. In contrast to our results on *B. glabrata* embryos, *Padina gymnospora* was active against *B. glabrata* adult snails [25]. Nevertheless, in our study, *P. tetrastomatica* killed 100% of the *B. glabrata* embryos at the blastulae stage. The molluscicidal activity of *Dictyota dichotoma* reported for *B. glabrata* snails [26] and *B. alexandrina* adult snails and eggs [27] agrees with our results for *D. ciliolata* and *D. mertensii*.

The most active extracts were obtained using non-polar extraction solvents in the present study, such as hexane, chloroform, dichloromethane, or supercritical CO_2_. Other studies have shown that polar solvents seem to be less efficient in obtaining molluscicidal compounds. The aqueous fractions of the dry methanol extracts of 60 seaweed species were screened for molluscicidal activity against *B. glabrata* at 500 ppm, and the majority of the extracts tested were inactive [26]. In another study, only two among eight seaweed aqueous suspensions had molluscicidal activity, even when tested at the high concentrations of 1000 and 5000 ppm [28].

### 2.2. Metabolomic Analysis

A recent study has demonstrated the efficacy of uni- and multivariate statistical analysis to highlight plant compounds such as ovicidal active against veterinary gastrointestinal nematodiasis [18]. Despite this, this strategy is still poorly explored in the search for anthelmintic and molluscicide activities, particularly in marine-based compounds. Thus, we applied this methodology for the first time to analyze alga extracts against *Biomphalaria glabrata* embryos and *Schistosoma mansoni*.

Due to the chemical diversity, the metabolomic analysis was performed with taxonomically related groups that presented at least three active extracts and three inactive/relatively inactive extracts. The correlation between the chemical composition of Ochrophyta extracts and their anthelmintic (worms) and molluscicidal (blastula) activities and the chemical composition of the species of *Laurencia* and *Laurenciella* sp. extracts and their molluscicidal (veliger) activity was established.

#### 2.2.1. *Laurencia/Laurenciella* Set

**Figure 1:**
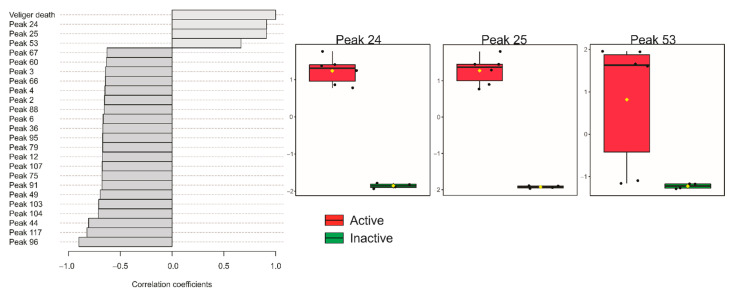
Pearson correlation pattern of the top 25 features from the 123 compounds detected in the GC–MS analysis of the extracts of *Laurencia aldingensis*, *Laurencia catarinensis*, *Laurencia dendroidea*, and *Laurenciella* sp. and the molluscicidal activity against *Biomphalaria glabrata* veliger embryos.

The pre-treatment of gas chromatography coupled to mass spectrometry (GC–MS) data provided 123 compounds (denominated peaks 1–123). Among them, peaks 24, 25, and 53 presented a statistically significant correlation with molluscicidal activity against *Biomphalaria glabrata* veliger embryos (Figure 1). Peaks 24 and 25 presented correlation coefficients higher than 0.91 (*p* < 0.001), being present in all active extracts, while peak 53 presented a correlation coefficient of 0.67 (*p* < 0.05) and was not detected in *L. aldingensis* hexanic extract or *Laurenciella* sp. methanolic extract. This pattern was confirmed by hierarchical cluster (Figure S1) and by partial least squares discriminant analysis (PLS-DA; Figure S2).

#### 2.2.2. Ochrophyta Set

**Figure 2:**
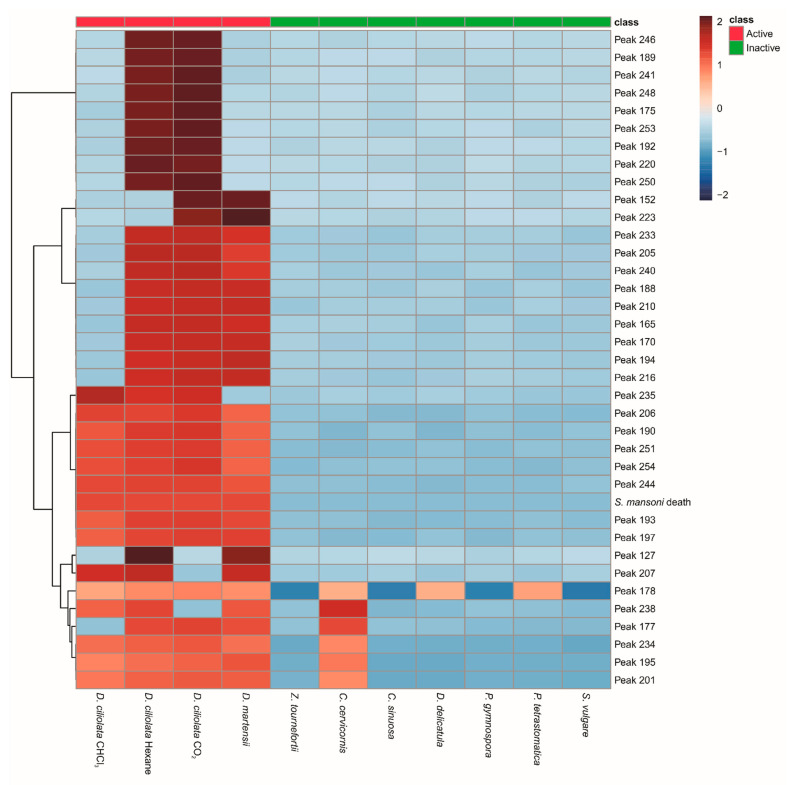
Heatmap of Ochrophyta compounds that exhibited a positive correlation with anthelmintic activity in the Pattern Hunter test.

**Figure 3:**
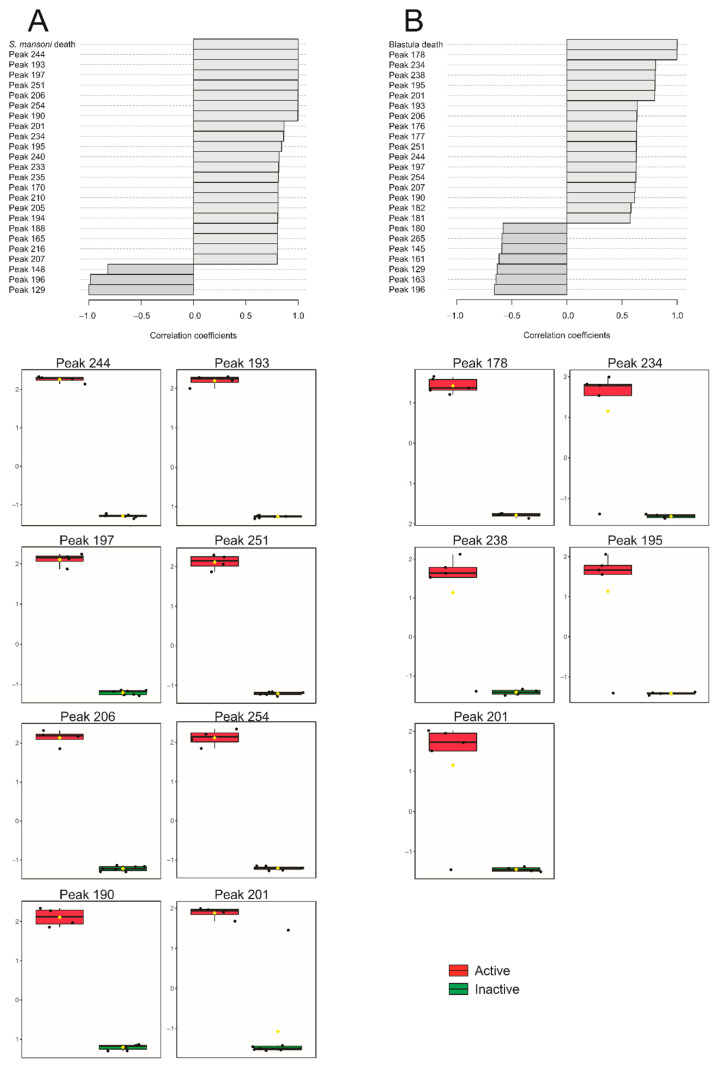
Pearson correlation pattern of the top 25 features from the 136 compounds (124–269) detected in the GC–MS analysis of the extracts of *Canistrocarpus cervicornis*, *Colpomenia sinuosa*, *Dictyota ciliolata*, *Dictyota mertensii*, *Padina gymnospora*, *Padina tetrastomatica*, *Sargassum vulgare*, and *Zonaria tournefortii;* (**A**) the anthelmintic activity against *Schistosoma mansoni* worms; (**B**) the molluscicidal activity against *Biomphalaria glabrata* blastula embryos.

In the GC–MS analysis of the extracts of Ochrophyta species, 136 compounds were found (denominated peaks 124–269). The correlation with anthelmintic activity pointed to 33 statistically significant peaks (Table 2). Of these, peaks 190, 193, 197, 206, 244, 251, and 254 presented the most significant correlation coefficient (r > 0.99, *p* < 0.0001), being considered the main hits detected in all active samples (Figure 2). The distribution of these compounds is represented in Figure 3A. Other peaks, such as 178, 195, 201, and 234, were also detected in all active samples, but they were found in the inactive *C. cervicornis* extract (Figure 2). Peaks 201 and 234 were found with lower intensity (near three times) than the lowest active extract, which leads us to consider them also as hits (Figure 2). On the other hand, compound 195 was detected with a higher intensity in *C. cervicornis* than in the *D. ciliolata* chloroform extract. Peak 178 was also found in the *D. delicatula* and *P. tetrastomatica* inactive extracts, with a higher intensity in *P. tetrastomatica* than in the *D. ciliolata* chloroform extract (Figure 2). In this way, these compounds cannot be considered hits for anthelmintic activity. These results were also observed in PLS-DA (Figure S3).

Peaks 165, 170, 188, 194, 205, 210, 216, 233, 235, and 240 were detected in three of four active extracts (r > 0.80, *p* < 0.005), being considered as secondary hits. Despite the detection of peak 177 in three active extracts, this compound could not be considered a hit once it was found in similar intensity in *C. cerviconis* (Figure 2). The peaks with a lower correlation (r > 0.60, *p* < 0.05): 127, 152, 175, 189, 192, 220, 223, 241, 246, 248, 250, and 253 were detected only in two active extracts and were suggested as tertiary hits for anthelmintic activity (Figure 2).

Regarding the molluscicidal activity against *Biomphalaria glabrata* blastula embryos, five peaks presented a correlation (Figure 3B). Only peak 178 was present in all active samples, presenting a correlation coefficient greater than 0.99 (*p* < 0.0001). Peaks 195, 201, 234, and 238 were detected in *C. cervicornis*, *D. ciliolata*, and *D. mertensii*. These patterns can also be seen in hierarchical cluster analyses (Figure S4) and PLS-DA (Figure S5).

The selection of taxonomically related species for the constitution of the sets for statistical analysis allowed for the alignment of the data despite the chemical complexity of the species studied. In this way, we were able to analyze the chemical compositions and biological activities’ interdependence and identify the most promising hits. The Pattern Hunter test was used to list these hits, and the heatmap permitted a closer look at the distribution pattern of these compounds in the extracts, especially in the analysis of schistosomicidal activity, contributing to an initial classification of the promising hits.

The selected hits were investigated on the basis of a literature comparison, previous knowledge from the chromatographic extract analysis, and a spectroscopic identification of isolated compounds [29,30,31]. More details on the peak annotation and mass fragmentation profile can be found in Supplementary Material (Table S1, Figures S6–S16).

Peaks 24 and 25 of the *Laurencia*/*Laurenciella* set indicate triquinane derivative sesquiterpenoids (Table 3). Two compounds of this class, silphiperfolan-7β-ol and 7-*epi*-silphiperfolan-6β-ol, have been reported as major constituents of the essential oil of *L. dendroidea* [29]. None of these compounds were previously indicated as molluscicidal or schistosomicidal, although a triquinane derivative from *L. dendroidea* has been reported against leishmaniasis [32].

Ochrophyta set analysis afforded nine major hits (Table 3). Seven of these peaks were putatively identified as diterpenes. The diterpenes of *Dictyota* are divided into three great groups: group I (prenylated guaianes), group II (dolastanes), and group III (xenianes and dichotomanes) [33,34]. In this way, peaks 190, 193, 201, 206, and 234 were suggested as dictyol derivatives (group I); peak 244 as 9-acetoxydichotoma-2,13-diene-16,17-dial, a dichotomane; and peak 251 as a xeniane derivative, both from group III. Some works have found compounds from the group I in the species of the present study, such as dictyol B, dictyol C, isopachydictyol A, and dictyotadiol [35,36]. 

Helminth infections remain an important health problem, mostly in developing countries, affecting over a billion people in sub-Saharan Africa, Asia, and the Americas [37]; nevertheless, efforts in anthelminthic drug discovery are still incipient. Only three new drug classes have reached the animal market since 2000, and no new anthelmintic classes have been approved for human use [38]. 

In the present study, standard biological models were used to screen for compounds of potential use in schistosomiasis control. A total of 22 of the 37 seaweed species screened were active in at least one of the two models, and the metabolomic analysis of the crude extracts pointed to several candidate drugs. 

The results reported in the present study come from a comprehensive preliminary screening, representing a compilation of data from material collected at different times over nine years. The active and inactive extracts were evaluated for their composition in order to correlate the chemical profile to biological activity regardless of the type of extraction. In this way, it was possible to submit the data from all the extracts to the statistical analysis. The information compiled here will be of fundamental importance for new research looking for bioactive seaweed metabolites. Additionally, the results obtained by using this innovative approach will decrease bottlenecks of bioguided studies.

## 3. Materials and Methods

### 3.1. Seaweed Samples and Extracts Preparation

The seaweed samples were collected in the intertidal zone from Espírito Santo State, Southeastern Brazil, and a voucher was deposited at the Maria Eneyda P. Kauffmann Fidalgo Herbarium (SP) at the Instituto de Botânica in São Paulo or herbarium VIES at the Universidade Federal do Espírito Santo.

After collection, the fresh and cleaned material was stored frozen in zip-lock plastic bags at −20 °C. Care was taken to remove sand particles and epiphytes. For extract preparation, dried and powdered algae were added to a single solvent (1:10 *w*/*v*) (dichloromethane or chloroform, depending on the availability) and allowed to soak overnight; this procedure was repeated 3 times. Alternatively, the dried and powdered algae were submitted to fractionated extraction, in which the sample was processed by 3 extraction cycles with hexane, followed by chloroform and then methanol. All obtained extracts were filtered and concentrated on a rotary evaporator. Supercritical CO_2_ was performed in Spe-ed SFE system, Applied Separations, at 45 °C, and a pressure of 280 bar with a CO_2_ flow of 12 mL/min for 1 h. 

### 3.2. Schistosomicidal Activity Screening

The life cycle of *S. mansoni* (Sambon, 1907) (Trematoda: Schistosomatidae) (BH strain—Belo Horizonte, MG, Brazil) was maintained in *Biomphalaria glabrata* (Say, 1818) (Gastropoda: Planorbidae) snails and *Mesocricetus auratus* (Waterhouse, 1839) (Mammalia: Cricetidae) hamsters. Female hamsters were infected by the injection of 300 cercariae subcutaneously and subjected to portal and mesenteric system perfusion for the recovery of *S. mansoni* adult worms 6 weeks later. The worms were washed in RPMI 1640 medium (Invitrogen), pH 7.5, supplemented with sodium bicarbonate (2000 µg/mL), penicillin (100 UI/mL), streptomycin (100 µg/mL), amphotericin B (0.25 µg/mL), and 10% fetal bovine serum (Gibco BRL). Adult male and female paired worms were transferred to 24-well culture plates with 1 mL of the medium per well. The seaweed extracts were dissolved in 1 mL of DMSO 1.5% (dimethyl sulfoxide) in RPMI medium and added to the cultured worms to achieve a final concentration of 100 µg/mL. The parasites were monitored after the first 2 h and then every 24 h for 96 h under a light microscope to evaluate the effects on motor activity and the mortality rate. A total absence of movement was the criterion for death. A 1.5% DMSO in RPMI 1640 solution was used in the negative control group, and praziquantel (PZQ) 4.8 µM (1.5 µg/mL) was used in the positive control group. The experiments were carried out in 5 replicates.

### 3.3. Molluscicidal Activity Screening in Biomphalaria Glabrata Embryos

*Biomphalaria glabrata* (Say, 1818) snails were originally from Barreiro de Baixo (Minas Gerais, Brazil) and have been maintained under laboratory conditions for more than 40 years in the Laboratory of Parasitology, Butantan Institute, São Paulo [39]. 

Plastic sheets were used as a substrate for oviposition. Embryos were observed by a stereomicroscope and for the selection of developmental stages. A minimum of 50 non-damaged embryos at the blastulae (0–15 h after the first egg cleavage) and veliger (96–111 h) stages were selected and maintained in Petri dishes with filtered and dechlorinated tap water until the exposure to extracts at a concentration of 100 mg/L for 24 h. All the egg masses were washed with dechlorinated tap water at the end of the exposure and observed daily for mortality and malformation effects for 7 days. Dechlorinated tap water was used in a negative control group, and 1 additional control group was maintained in 1.5% DMSO under the same experimental conditions. Activity assays on embryos were conducted at 25 ± 2 °C with a 12-h light period. The assays were performed according to the World Health Organization procedure [40,41], and experimental procedures were employed according to accepted principles of animal welfare in experimental science (CEUA N 5042140818). 

### 3.4. Gas Chromatography–Mass Spectrometry (GC–MS) Analysis

Gas chromatography coupled with mass spectrometry (GCMS-QP2010 Plus, Shimadzu, Japan) was used to analyze the extracts.

The chromatographic conditions were performed in a HP-5MS capillary column (30 m × 0.25 mm × 0.1 μm), and helium was used as carrier gas (with a constant flow rate of 1 mL/min). The oven temperature was increased at 3 °C/min from 60 to 260 °C and held for 40 min. The injection and transfer line temperatures were 220 and 240 °C. The MS system temperatures of the ion source was maintained at 240 °C, and the electron impact ionization was employed with a collision energy of 70 eV. The detection was performed in the full scan mode with a mass range of 50–1000 *m/z*. The target compounds were identified by the literature comparison and previous knowledge about their mass spectra.

### 3.5. Sample Selection for Statistical Analysis

The analysis was performed for 2 sets of species: the *Laurencia*/*Laurenciella* set and the Ochrophyta set. The first set was composed of the extracts of *L. aldingensis* (hexane, chloroform, and methanol), *L. catarinensis* (chloroform), *L. dendroidea* (hexane, chloroform, and methanol), and *Laurenciella* sp. (hexane, chloroform, and methanol). The species were divided into 2 groups (active and inactive) considering their molluscicidal activity against veliger embryos.

The second set was composed of the extracts of *C. cervicornis* (dichloromethane), *C. sinuosa* (chloroform), *D. ciliolata* (hexane, chloroform, and supercritical fluid), *D. mertensii* (supercritical fluid), *P. gymnospora* (chloroform), *P. tetrastomatica* (chloroform), *S. vulgare* (chloroform), and *Z. tournefortii* (chloroform). The chemical data of these extracts were correlated with 2 activities: anthelmintic activity against *S. mansoni* worms and molluscicidal activity against blastula embryos. *D. ciliolata* supercritical fluid extract was excluded from molluscicidal analysis since it was not tested for this activity.

### 3.6. Data Processing, Correlation Analysis, and Compound Identification

Raw data obtained from the GCMSsolution^®^ software were transformed into CDF format. The ion peaks with an intensity greater than 2000 were aligned using MetAlign software [42], resulting in 19,790 entrances for the *Laurencia*/*Laurenciella* set and 3147 for the Ochrophyta set. The entries were grouped into 123 (denominated peaks 1–123) and 136 reconstituted compounds (denominated peaks 124–269), respectively, using MSClust [43]. The percentages of *S. mansoni* worm, blastula, or veliger embryo death were added as entrances to the respective exported data. The matrix was uploaded to the MetaboAnalyst 4.0 platform [44], where they were log-transformed, Pareto scaled, and submitted to statistical analysis.

The Pattern Hunter tool was used to apply Pearson’s correlations (significant at *p* ≤ 0.05) to the chemical composition, and biological activity was determined. Hierarchical clustering analysis (HCA) and partial least squares discriminant analysis (PLS-DA) were used to observe the metabolite distribution in the function of the biological activities.

Compound identification was performed on the basis of the EI-MS spectra by comparing these data with information reported in the literature.

## Figures and Tables

**Table 1:** Schistosomicidal effect of Brazilian seaweed extracts on *Schistosoma mansoni* worms and *Biomphalaria glabrata* embryos.

Algae Species	Extract	*Schistosoma mansoni*					*Biomphalaria glabrata embryos*	
		Death Ratio (%)			Couple Separation (%)	Eggs (Average)	Death Ratio (%)	
		Female	Male	Total			*Blastula*	Veliger
**Rhodophyta**								
* Amphiroa fragilissima*	Chloroform	0	0	0	80	40	100	0
* Bostrychia tennela*	Dichlorometane	0	80	40	100	69	0	0
* Botryocladia occidentalis*	Dichlorometane	0	0	0	0	379	100	0
* Bryothamnion seaforthii*	Chloroform	0	0	0	0	188	0	0
* Ceratodictyon variabile*	Dichlorometane	0	0	0	0	186	0	0
* Cryptonenia crenulata*	Chloroform	0	0	0	100	108	100	0
* Cryptonenia seminervis*	Chloroform	0	0	0	20	157	100	0
* Dichotomaria marginata*	Chloroform	0	0	0	100	0	100	0
*Gracilaria* cf. *intermedia*	Chloroform	0	0	0	0	301	0	0
* Gracilaria domingensis*	Chloroform	0	0	0	40	47	1	5
* Hypnea nigrescens*	Chloroform	0	0	0	20	223	0	0
* Jania rubens*	Chloroform	0	0	0	100	25	100	0
* Laurencia aldingensis*	Chloroform	0	40	20	100	0	100	11
* Laurencia aldingensis*	Methanol	0	0	0	0	190	57	2
* Laurencia aldingensis*	Hexane	100	100	100	80	0	100	100
* Laurencia catarinensis*	Chloroform	0	0	0	100	0	100	21
* Laurencia dendroidea*	Hexane	100	100	100	80	0	100	100
* Laurencia dendroidea*	Chloroform	100	100	100	100	0	100	100
* Laurencia dendroidea*	Methanol	0	0	0	60	116	100	100
*Laurenciella* sp.	Hexane	0	0	0	100	2	100	100
*Laurenciella* sp.	Chloroform	100	100	100	100	19	71	6
*Laurenciella* sp.	Methanol	0	0	0	20	200	100	70
* Octhodes secundiramea*	Chloroform	100	100	100	100	0	100	100
* Palisada perforata*	Chloroform	0	0	0	0	291	0	0
* Palisada flagellifera*	Dichlorometane	0	0	0	0	149	100	0
* Porphyra spiralis*	Chloroform	100	100	100	100	37	6	0
* Pterocladiella capillacea*	Chloroform	0	0	0	0	191	-	-
* Solieria filiformis*	Chloroform	0	0	0	100	0	100	0
* Spyridia aculeata*	Chloroform	0	0	0	100	35	100	100
* Tricleocarpa cylindrica*	Chloroform	0	0	0	100	13	100	0
*Vidalia obtusiloba*	Chloroform	0	0	0	100	74	56	0
**Ochrophyta**								
*Canistrocarpus cervicornis*	Dichlorometane	0	0	0	100	0	100	0
* Colpomenia sinuosa*	Chloroform	0	0	0	0	89	0	0
* Dictyota ciliolata*	Hexane	100	100	100	100	1	100	11
* Dictyota ciliolata*	Chloroform	100	100	100	100	0	100	40
* Dictyota ciliolata*	Supercritical fluid	100	100	100	100	0	-	-
* Dictyota mertensii*	Supercritical fluid	100	100	100	100	0	100	10
* Padina gymnospora*	Chloroform	0	0	0	0	284	0	0
* Padina tetrastomatica*	Chloroform	0	0	0	0	306	100	0
* Sargassum vulgare*	Chloroform	0	0	0	20	120	0	0
*Zonaria tournefortii*	Chloroform	0	0	0	0	168	0	0
**Chlorophyta**								
* Caulerpa cupressoides*	Chloroform	0	0	0	0	211	0	0
* Caulerpa racemosa*	Chloroform	0	0	0	80	12	100	0
* Caulerpa sertularioides*	Chloroform	0	0	0	100	4	0	0
*Codium isthmocladum*	Chloroform	0	20	10	100	108	100	0
**Controls**								
PQZ (positive control)		40	100	80	0	0	-	-
DMSO (negative control)		0	0	0	0	266	0	0

PZQ: praziquantel; DMSO: dimethyl sulfoxide.

**Table 2:** Pearson correlation between all significative compounds from the 136 peaks (124–269) detected in the GC–MS analysis of the extracts of *Canistrocarpus cervicornis*, *Colpomenia sinuosa*, *Dictyota ciliolata*, *Dictyota mertensii*, *Padina gymnospora*, *Padina tetrastomatica*, *Sargassum vulgare*, and *Zonaria tournefortii* and the anthelmintic activity against *Schistosoma mansoni* worms and the molluscicidal activity against *Biomphalaria glabrata* blastula embryos.

Peak	*S. mansoni*		Blastula	
	Pearson Correlation	*p*-Value	Pearson Correlation	*p*-Value
127	0.61809	0.042685	-	-
152	0.60424	0.048964	-	-
165	0.80143	0.003019	-	-
170	0.80758	0.002645	-	-
175	0.60873	0.04686	-	-
177	0.61303	0.04491	-	-
178	0.63281	0.036648	0.99671	<0.00001
188	0.80212	0.002975	-	-
189	0.61548	0.043823	-	-
190	0.99667	<0.00001	-	-
192	0.61426	0.044361	-	-
193	0.99896	<0.00001	-	-
194	0.80438	0.002835	-	-
195	0.84093	0.001182	0.7983	0.009899
197	0.99821	<0.00001	-	-
201	0.86399	0.000605	0.79584	0.010302
205	0.80558	0.002762	-	-
206	0.9975	<0.00001	-	-
207	0.79946	0.003146	-	-
210	0.8075	0.00265	-	-
216	0.80123	0.003031	-	-
220	0.62674	0.039061	-	-
223	0.61416	0.044408	-	-
233	0.81422	0.002282	-	-
234	0.86116	0.000661	0.80394	0.009017
235	0.81016	0.002499	-	-
238	-	-	0.79904	0.009781
240	0.8183	0.002077	-	-
241	0.61611	0.043545	-	-
244	0.9995	<0.00001	-	-
246	0.60917	0.046658	-	-
248	0.62201	0.041017	-	-
250	0.62764	0.038698	-	-
251	0.99785	<0.00001	-	-
253	0.62485	0.039835	-	-
254	0.99737	<0.00001	-	-

**Table 3:** Candidate peaks with the most significant correlation coefficient on active samples for treating schistosomiasis and the indicated chemical skeleton suggestion based on the literature.

Peak	Anotation			Retention Time (min)
	Class	Sub-Class	Compound	
***Laurencia/Laurenciella* set**				
24	Sesquiterpene	Triquinane alcohol	Silphiperfolanol derivative	27.39
25	Sesquiterpene	Triquinane alcohol	Silphiperfolan-7β-ol (C_15_H_26_O)	27.41
53	Unknown	-	-	43.98
Ochrophyta set				
190	Diterpene	Prenylated guaiane (Group I)	Dictyol derivative	51.53
193	Diterpene	Prenylated guaiane (Group I)	Dictyol derivative	52.49
197	Unknown	-	-	53.37
201	Diterpene	Prenylated guaiane (Group I)	Dictyol derivative	54.15
206	Diterpene	Prenylated guaiane (Group I)	Dictyol derivative	54.91
234	Diterpene	Prenylated guaiane (Group I)	Dictyol derivative	59.17
244	Diterpene	Dichotomane (Group III)	9-Acetoxydichotoma-2,13-diene-16,17-dial (C_22_H_32_O_4_)	60.87
251	Diterpene	Xeniane (Group III)	Xeniane derivative	62.44
254	Unknown	-	-	62.90

## Data Availability

Not applicable.

## Supplementary Materials

The following are available online at https://www.mdpi.com/article/10.3390/md19050234/s1, Table S1: Mass fragmentation pattern for *Laurencia/Laurenciella* set, and *Dictyota* set selected peaks and literature data. Figure S1: Heatmap of the top 25 features from the 123 compounds detected in the GC–MS analysis of the extracts of *Laurencia aldingensis*, *Laurencia catarinensis*, *Laurencia dendroidea*, and *Laurenciella* sp. and the molluscicidal activity against *Biomphalaria glabrata* veliger embryos. Figure S2: PLS-DA analysis considering the 123 compounds detected in the GC–MS analysis of the extracts of *Laurencia aldingensis*, *Laurencia catarinensis*, *Laurencia dendroidea*, and *Laurenciella* sp. and the molluscicidal activity against *Biomphalaria glabrata* veliger embryos. Figure S3: PLS-DA analysis considering the 136 compounds detected in the GC–MS analysis of the extracts of Ochrophyta and the anthelmintic activity against *Schistosoma mansoni* blastula embryos. Figure S4: Heatmap of the top 50 features from the 136 compounds detected in the GC–MS analysis of the extracts of Ochrophyta and the molluscicidal activity against *Biomphalaria glabrata* blastula embryos. Figure S5: PLS-DA analysis considering the 136 compounds detected in the GC–MS analysis of the extracts of Ochrophyta and the molluscicidal activity against *Biomphalaria glabrata* blastula embryos. Figure S6: Terpene skeleton from *Laurencia/Laurenciella* (A) and *Dictyota* (B) with potential for treating schistosomiasis based on the metabolomic analysis. Figure S7: Peak 24. Figure S8: Peak 25. Figure S9: Peak 53. Figure S10: Peak 234. Figure S11: Peak 190. Figure S12: Peak 201. Figure S13: Peak 193. Figure S14: Peak 206. Figure S15: Peak 244. Figure S16: Peak 251.
