# Supplementary material for: The Prospective Use of Brazilian Marine Macroalgae in Schistosomiasis Control
Source: Mar Drugs. 2021 Apr 22;19(5):234. doi: 10.3390/md19050234 (PMC8143572; doi:10.3390/md19050234)
Supplement: Supplementary file 1 [file marinedrugs-19-00234-s001.zip › marinedrugs-1164973-supplementary.pdf]

SUPPLEMENTARY MATERIAL

Supplementary Table S1

Mass fragmentation pattern for *Laurencia* and *Laurenciella* set, and *Dictyota* set selected peaks and literature data.

Peak	Compound	RT (min)	$EIMS_{Ob}$ (70 eV) *m/z* (rel. int) [a]	$EIMS_{Lit}$ (70 eV) *m/z* (rel. int)	References
***Laurencia* and *Laurenciella* set**					
24	**SESQUITERPENE** **Triquinane alcohol** Silphiperfolanol derivative	27.39	222 (2), 204 (5), 189 (3), 175 (10), 164 (6), 135 (38), 122 (10), 123 (11), 111 (12), 109 (20), 107 (23), 105 (7), 98 (100), 93 (16), 83 (32), 79 (11), 67 (9), 55 (11), 43 (17)	7-epi-silphiperfolan-6β-ol: $C_{15}H_{26}O$, 222 (2), 203 (4), 201 (15), 175 (12), 164 (10), 149(13), 145 (9), 135 (75), 133(12), 111 (15), 109 (33), 107 (30), 105 (20), 98 (100), 95 (52), 91 (23)	Wright et al. 1990 [b]
				7-epi-silphiperfolan-6β-ol: $C_{15}H_{26}O$, 222 (2), 204 (4), 189 (4), 175 (14), 164 (12), 149 (10), 136 (16), 135 (98), 122 (11), 109 (20), 107 (20), 98 (100), 95 (40), 93 (20), 91 (14), 83 (32), 81 (20), 79 (16), 67 (14), 55 (18)	Weyerstahl et al. 1998 [d]
				7-epi-silphiperfolan-6β-ol: $C_{15}H_{26}O$, 222 (1); 204 (8); 189 (4); 175 (19); 164 (13); 136 (17); 135 (95); 122 (12); 109 (22); 107 (27); 98 (100); 95 (41); 93 (22); 91 (12); 83 (35); 81 (20); 79 (15); 67 (13); 55 (13); 43 (32); 41 (12)	Stein, EM (in-house library)[a]
25	**SESQUITERPENE** **Triquinane alcohol** silphiperfolan-7β-ol ($C_{15}H_{26}O$)	27.41	222 (1), 204 (1), 189 (1), 175 (2), 165 (2), 151 (2), 135 (22), 125 (5), 121 (10), 107 (17), 95 (29), 86 (100), 81 (41), 67 (10), 55 (10), 43 (19)	222 (2), 207 (1), 204 (1), 189 (2), 175 (2), 165 (1), 161 (1), 151 (2), 149 (2), 137 (14), 136 (11), 135 (28), 125 (6), 121 (7), 107 (9), 95 (21), 93 (8), 86 (100), 85 (16), 81 (35), 79 (8), 71 (7), 67 (10), 55 (12);	Weyerstahl et al. 1998 [d]
				222 (2); 204 (1); 189 (1); 175 (1); 165 (1), 147 (1); 137 (11); 136 (11); 135 (28); 125 (5); 121 (7); 107 (9); 95 (20); 93 (8); 91 (6); 86 (100); 85 (15); 81 (28); 79 (8); 67 (7); 55 (8); 43 (21)	Gressler 2011 [a]

53	Unknown	43.98	117 (40), 116 (6), 115 (33), 105 (29), 104 (31), 103 (30), 95 (17), 93 (36), 92 (18), 91 (100), 81 (22), 80 (21), 79 (64), 78 (45), 77 (45), 69 (24), 67 (39), 66 (6), 65 (44), 55 (18), 53 (20), 51 (13), 43 (2), 41 (24)	n.d.	n.d.
***Dictyota* set**					
190	**DITERPENE (Group I)** **Prenylated guaiane** Dictyol Derivative	51.53	304 (3.8); 286 (3.2); 243 (2.4); 223 (5.5); 215 (1.7); 206 (5.8); 181 (40.3); 173 (4.5); 175 (8.0); 157 (4); 142 (1.3); 143 (2.7); 129 (3.2); 125 (48.6); 123 (74); 109 (47.6); 107 (47.1); 105 (28.9); 95 (44.6); 93 (45.2); 91 (24.5); 81 (68.0); 79 (25.9); 69 (68.9); 43 (100); 41 (46.6)	Dictyol B: $C_{20}H_{32}O_2$, 304 (1); 286 (50); 268 (10); 253 (4); 225 (9); 215 (9); 197 (22); 186 (22); 173 (28); 157 (100); 155 (9); 142 (27); 129 (29); 105 (30); 91 (35); 79 (27); 69 (45); 55 (29); 41 (45)	Freitas et al. 2007 [e]
193	**DITERPENE (Group I)** **Prenylated guaiane** Dictyol Derivative	52.49	288 (6.2); 270 (6.7); 255 (12.4); 245 (4.5); 230 (23.2); 227 (13.2); 215 (16.9); 201 (10.6); 188 (14.5); 173 (71.3); 159 (30.2); 148 (45.9); 147 (31.5); 145 (39.1); 135 (48.8); 133 (61.5); 123 (44.4); 122 (40.6); 121 (50.5); 119 (53.9); 109 (52.5); 107 (78.6); 105 (49.8); 95 (55.7); 93 (71.9); 91 (34.2); 81 (63.8); 79 (41.3); 69 (38.4); 67 (41.8); 59 (67.6); 55 (43.8); 43 (100); 41 (33.6)	Isopachydictyol: $C_{20}H_{32}O$, 288 (12), 270 (6); 255 (7); 227 (4); 188 (20); 173 (20); 159 (96); 145 (42); 119 (70); 107 (59); 91 (61); 69 (71); 55 (61); 41 (100)<br>Isopachydictyol: $C_{20}H_{32}O$, 288 (10), 270 (8), 255 (5), 159 (80), 145 (30), 120 (40), 107 (60), 105 (80), 82 (100), 69 (100), 55 (90)	Cavalcanti 2004 [e], Cavalcanti et al. 2006 [e], Freitas et al. 2007 [e]<br>Ayyad et al. 2011[f]
197	Unknown	53.37	286 (2.5); 271 (2.2); 257 (2.2); 243 (4.2); 230 (1.9). 215 (9.1). 203 (10.9); 187 (7.5); 175 (13.6); 159 (17.1); 147 (43.4); 145 (26.3); 133 (49.7); 121 (36.1); 119 (51.2); 109 (40.1); 107 (55); 105 (73.8); 93 (50.2); 91 (56.6); 81 (52.6); 79 (42); 77 (23.8); 69 (100); 55 (56.7); 41 (78.1)	n.d.	n.d.

201	**DITERPENE (Group I)**<br>**Prenylated guaiane**<br>Dictyol Derivative	54.15	306 (1.7); 288 (27); 270 (8.4); 255 (29.9); 245 (4.1); 230 (5.1); 217 (9.2); 203 (21.0); 185 (38.2); 177 (29.9); 159 (85.5); 145 (29.7); 135 (29.8); 133 (29.3); 131 (31.6); 121 (43.2); 120 (32.8); 119 (100); 109 (44); 107 (71.7); 105 (43.7); 95 (33.5); 93 (44); 91 (34.8); 81 (66.3); 79 (37.5); 69 (78.2); 55 (44.1); 43 (75.7); 41 (59)	Dictyol C: $C_{20}H_{34}O_2$, 306 (5); 288 (37); 270 (12); 255 (28); 245 (6); 203 (27); 185 (25); 177 (39); 159 (69); 145 (22); 121 (37); 119 (72); 81 (53); 69 (78); 41 (100)<br>Dictyol C: $C_{20}H_{34}O_2$, 306 (2), 288 (18), 270 (49), 177 (71), 175 (51), 159 (47), 69 (100)	De-Paula et al. 2018 [e], Cavalcanti et al. 2006 [e], Freitas et al. 2007 [e]<br>Danise et al. 1977 [n.i.]
206	**DITERPENE (Group I)**<br>**Prenylated guaiane**<br>Dictyol Derivative	54.91	304 (3.4); 288 (1.8); 286 (1.9); 271 (0.3); 257 (6.8); 243 (0.3); 225 (3.0); 219 (15); 201 (5.7); 187 (3.5); 175 (13.5); 173 (13.6); 159 (12.1); 147 (24.4); 145 (18.6); 135 (14.6); 133 (15); 131 (15.5); 121 (16.4); 119 (18.6); 109 (30.6); 107 (23.8); 105 (27.9); 95 (27.6); 93 (25.5); 91 (26.4); 83 (16.1); 82 (82.6); 81 (60.5); 80 (100); 79 (34.5); 77 (15.4); 69 (57.6); 67 (29.6); 55 (33.9); 41 (50.8).	Dictyotadiol: $C_{20}H_{32}O_2$, 304 (5); 286 (19); 271 (12); 253 (6); 243 (3); 219 (13); 201 (39); 187 (17); 175 (21); 159 (20); 133 (27); 121 (25); 109 (44); 95 (40); 82 (100); 69 (45); 55 (44); 41 (70)<br>Dictyotadiol: $C_{20}H_{32}O_2$, 304, 286, 271, 243, 219, 201, 187, 175, 159, 133, 121, 109, 95, 82 (base peak), 69), 55.	Cavalcanti et al. 2006 [e]<br>De-Paula et al. 2008 [e]
234	**DITERPENE (Group I)**<br>**Prenylated guaiane**<br>Dictyol Derivative	59.17	302 (2); 284 (4.4); 269 (3.4), 255 (3.6); 201 (22.7); 159 (31.3); 145 (55.3); 135 (24.3); 133 (26.9); 131 (33.5); 121 (25.5); 119 (34.8); 117 (23); 109 (45.8); 107 (41.1) 105 (43.8); 95 (33.3); 93 (33); 91 (37.4); 81 (41.7); 79 (29.9); 69 (100); 67 (28.8); 55 (44.6); 43 (83.7); 41 (59.8)	Dictyol A: $C_{20}H_{30}O_2$, 302, 284, 273, 269, 255	Fattorusso et al. 1976 (n.i)
244	**DITERPENE (Group III)**<br>**Dichotomane**<br>9-Acetoxydichotoma-2,13-diene-16,17-dial ($C_{22}H_{32}O_4$)	60.87	342 (0.6); 327 (0.1); 300 (2.2); 282 (6.1); 267 (6.2); 239 (8.8); 189 (13.6); 171 (11.3); 161 (24.1); 145 (24.3); 135 (27.8); 133 (25.3); 119 (25.4); 109 (53.5); 107 (29.8); 105 (29.3); 95 (30.4); 93 (28.1); 91 (32); 82 (26.1); 81 (45.9); 69 (97.4); 67 (26.6); 55 (44.8); 43 (100); 41 (63.2)	342 (7); 327 (1); 300 (9); 282 (12); 267 (10); 239 (15); 189 (24); 161 (24); 149 (30); 109 (45); 91 (40); 69 (68); 43 (100)	Ortis-Ramires et al. 2008 [e], Cavalcanti 2004 [e], Cavalcanti et al. 2006, 2010 [e]

251	**DITERPENE (Group III)** **Xeniane** Xeniane derivative	62.44	360 (2.5); 318 (35.3); 300 (3.3); 285 (4.9); 283 (0.5), 282 (0.9); 275 (32.5); 243 (5.1); 229 (10.7); 219 (22.2): 201 (12.4); 189 (25.8); 177 (12.0); 176 (6.4); 175 (14.3); 173 (15.2); 161 (21.5); 147 (22.3); 145 (31); 135 (18.3); 133 (25.4); 119 (25.7); 109 (57.6); 105 (32.3); 95 (26.2); 91 (33); 82 (47.9); 69 (100); 67 (31.1); 55 (47.8); 43 (93.2); 41 (69.8)	4β-Acetoxydictyodial $C_{22}H_{32}O_4$ (**11**): 360 (8); 300 (9); 282 (n.i.); 201(15); 189 (38); 177 (16); 176 (15); 161 (26); 147 (18); 135 (9); 109 (47); 95 (24); 93 (20); 91 (34); 69 (92). 55 (42), 43 (100)	De-Paula et al. 2018 [e], De-Paula et al. 2008 [e]
254	unknown	62.90	358 (1); 300 (6); 282 (2); 269 (2); 253 (2); 237 (3); 218 (22); 171 (23); 157 (19); 145 (21); 143 (340); 131 (24); 129 (19); 119 (19); 109 (50); 107 (21); 105 (25); 95 (22); 93 (20); 91 (31); 82 (100); 81 (23); 79 (23); 69 (67), 67 (32); 55 (33); 44 (29); 43 (80); 41 (52)	n.d.	n.d.

[a] QP2010 Plus (Shimadzu), HP-5MS capillary column; [b] JEOL D-100; [c] Agilent 6890N chromatograph with an Agilent 50973 mass analyzer, [d] GC-MS: Hewlett Packard HP 5890 II (12.5 m HP-1 column) combined with MSD 5971 A; [e] HRGC-MS on a HP6890 series GC system (Agilent), HP 5973 mass selective detector, HP-1MS capillary column; [f] Kratos MS-25 instrument. n.i.: Not informed data; n.d.: not determined

Supplementary Figure S1

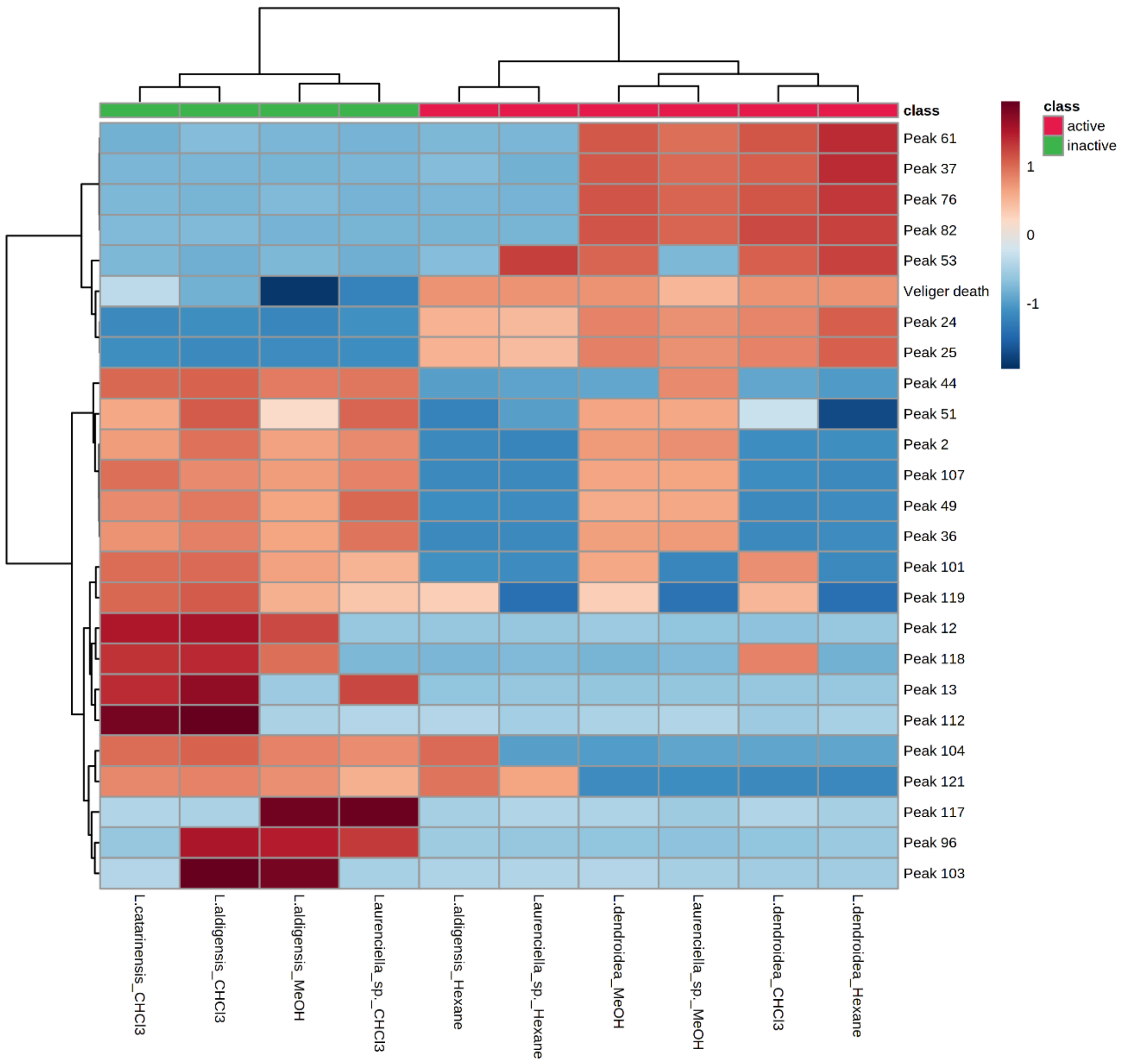

Heatmap of the top 25 features from the 123 compounds detected in the GC-MS analysis of the extracts of *Laurencia aldingensis*, *Laurencia catarinensis*, *Laurencia dendroidea*, *Laurenciella* sp. and the molluscicidal activity against *Biomphalaria glabrata* veliger embryos.

Supplementary Figure S2

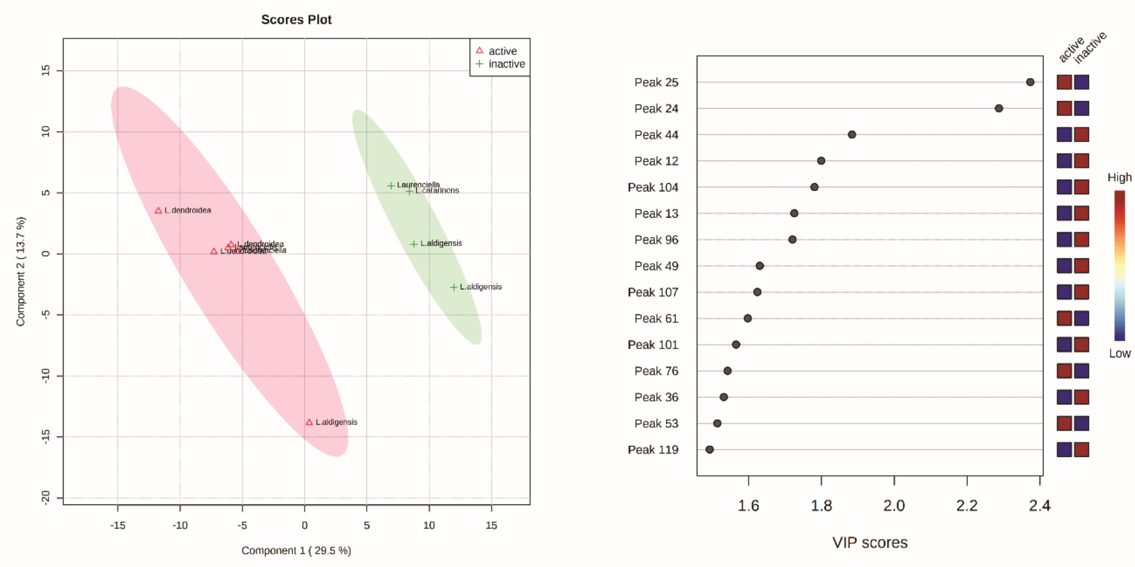

PLS-DA analysis considering the 123 compounds detected in the GC-MS analysis of the extracts of *Laurencia aldingensis*, *Laurencia catarinensis*, *Laurencia dendroidea*, *Laurenciella* sp. and the molluscicidal activity against *Biomphalaria glabrata* veliger embryos.

Supplementary Figure S3

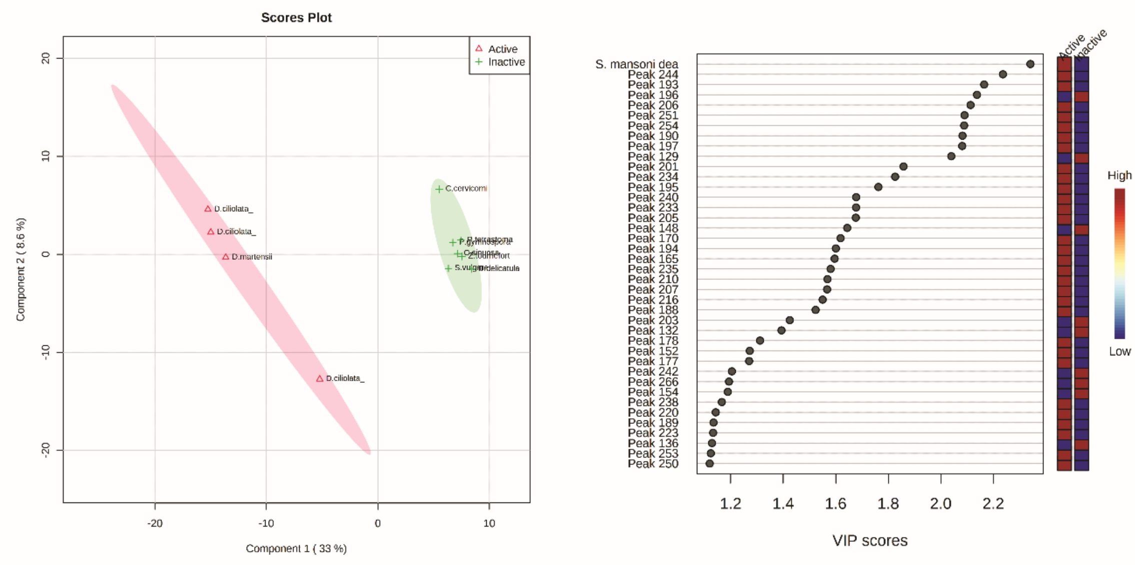

PLS-DA analysis considering the 136 compounds detected in the GC-MS analysis of the extracts of Ochrophyta and the anthelmintic activity against *Schistosoma mansoni* blastula embryos.

Supplementary Figure S4

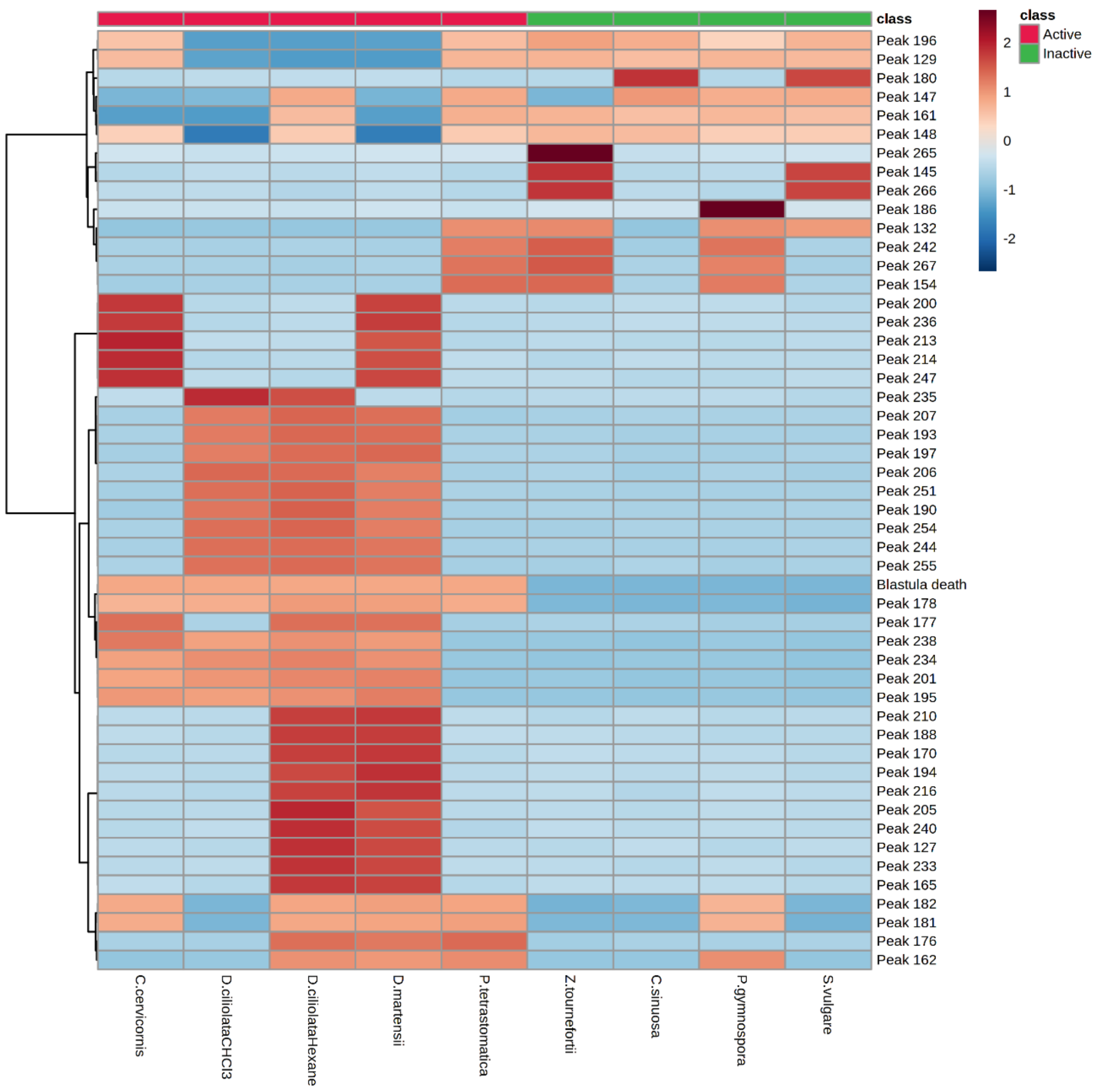

Heatmap of the top 50 features from the 136 compounds detected in the GC-MS analysis of the extracts of Ochrophyta and the molluscicidal activity against *Biomphalaria glabrata* blastula embryos.

Supplementary Figure S5

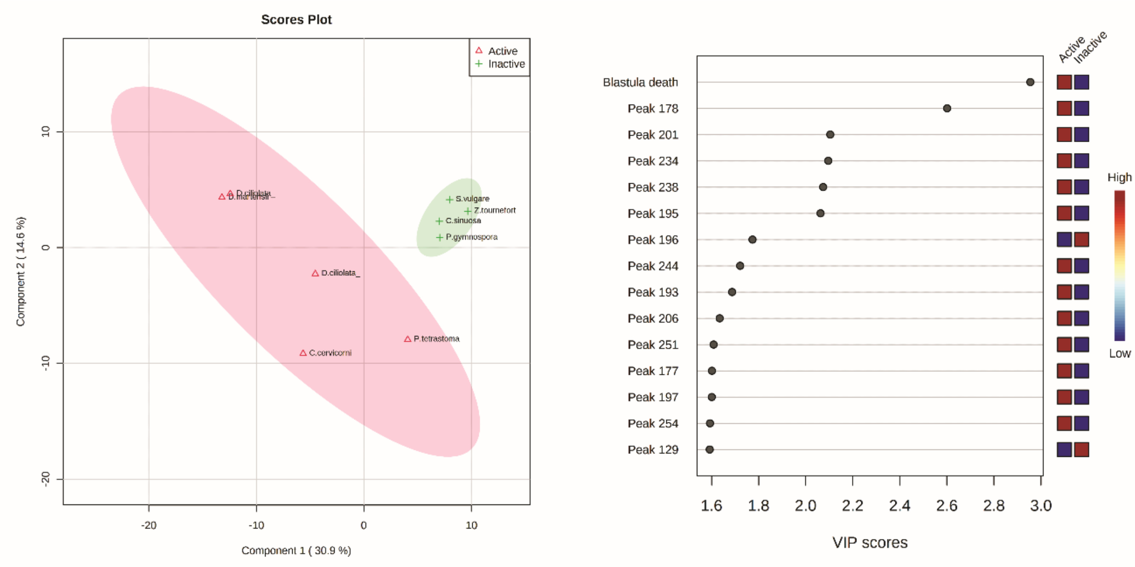

PLS-DA analysis considering the 136 compounds detected in the GC-MS analysis of the extracts of Ochrophyta and the molluscicidal activity against *Biomphalaria glabrata* blastula embryos.

Supplementary Figure S6

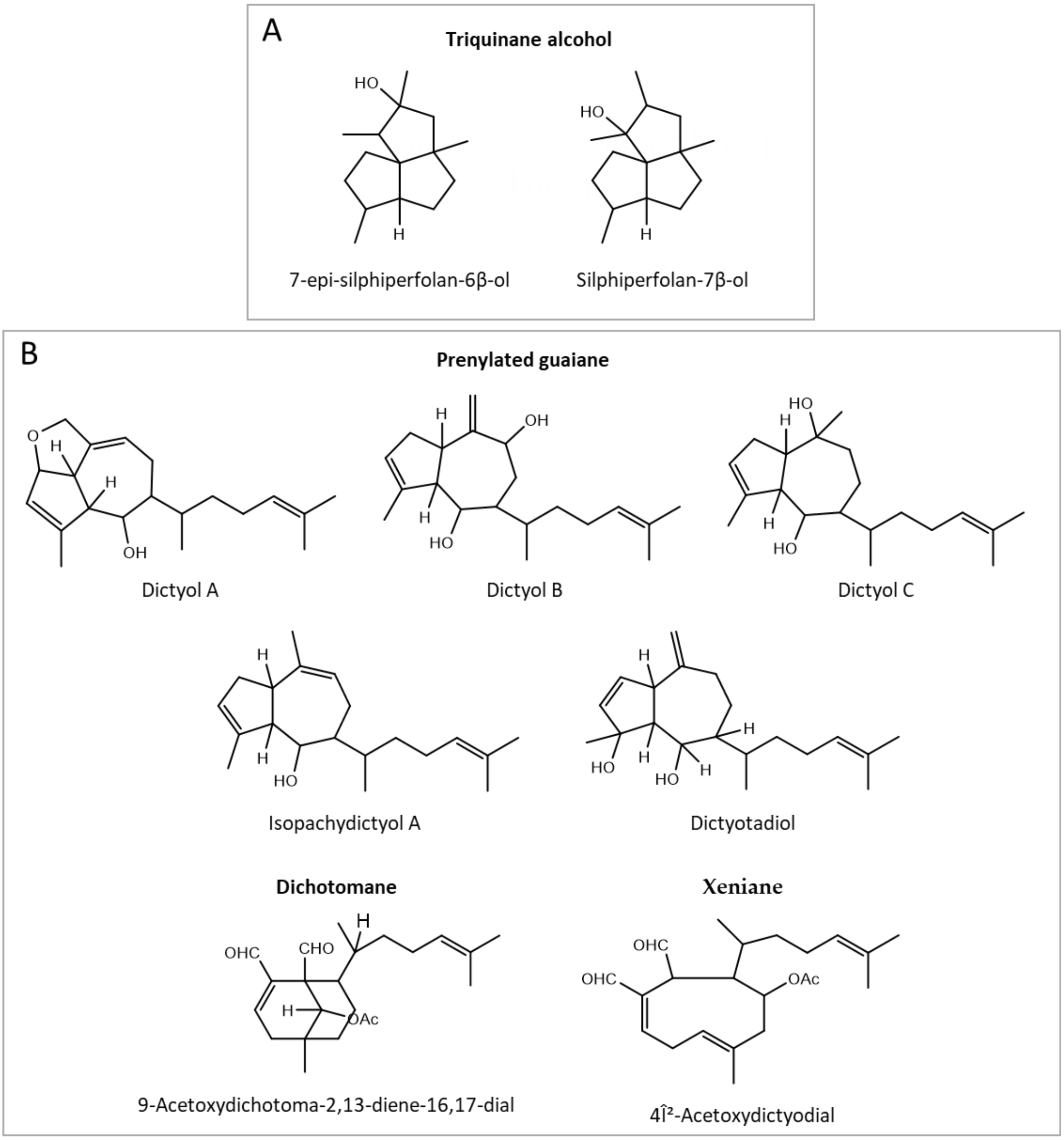

Terpene skeleton from *Laurencia*/*Laurenciella* (A) and *Dictyota* (B) with potential for treating schistosomiasis based on the metabolomic analysis.

MASS SPECTRA – SAMPLES AND LITERATURE

Supplementary Figure S7: peak 24

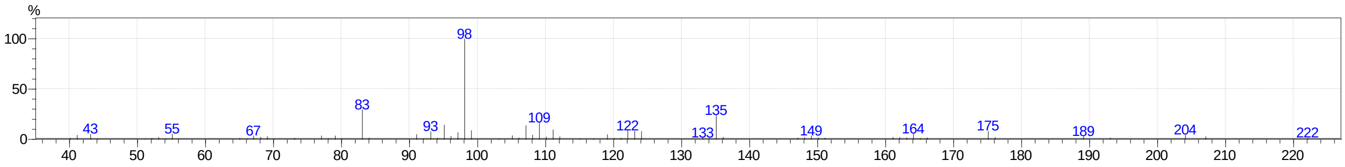

Peak 24 analyzed by gas chromatograph coupled to a mass spectrometer (GCMS-QP2010 Plus, Shimadzu, Japan) with HP-5MS capillary column (present study).

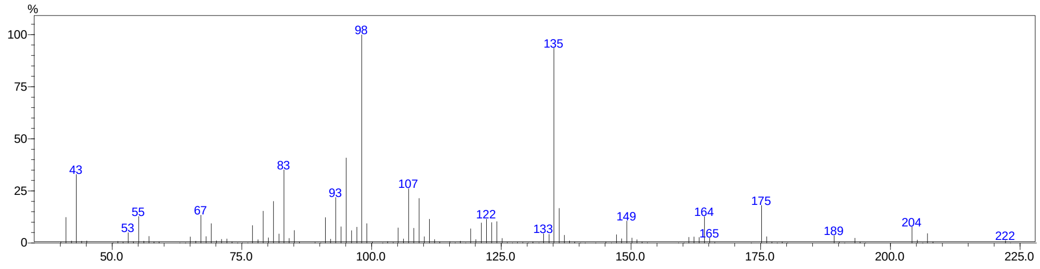

7-*epi*-silphiperfolan-6β-ol (1) analyzed by gas chromatograph coupled to a mass spectrometer (GCMS-QP2010 Plus, Shimadzu, Japan) with HP-5MS capillary column (in-house library)

Supplementary Figure S8: peak 25

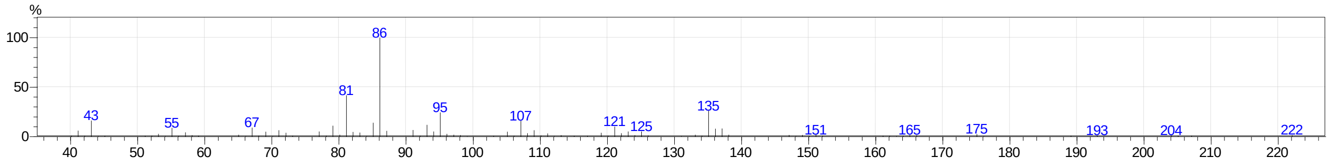

Peak 25 analyzed by gas chromatograph coupled to a mass spectrometer (GCMS-QP2010 Plus, Shimadzu, Japan) with HP-5MS capillary column (present study).

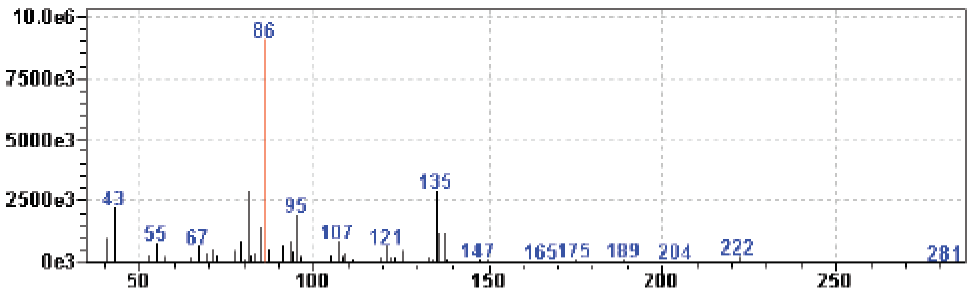

Silphiperfolan-7β-ol analyzed by gas chromatograph coupled to a mass spectrometer (GCMS-QP2010 Plus, Shimadzu, Japan) with Rtx-5MS capillary column (Gressler et al. 2011)

Supplementary Figure S9: peak 53

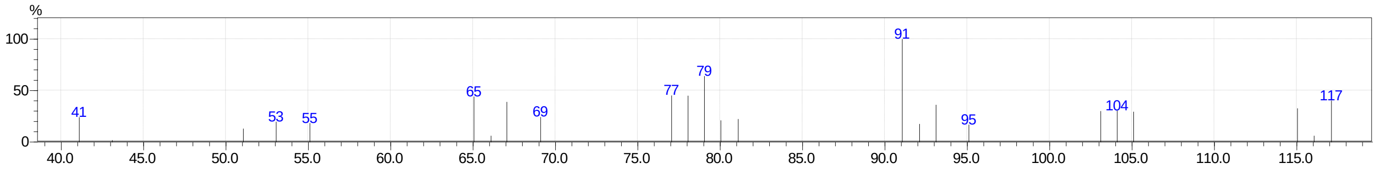

Peak 53 analyzed by gas chromatograph coupled to a mass spectrometer (GCMS-QP2010 Plus, Shimadzu, Japan) with HP-5MS capillary column (present study).

Supplementary Figure S10: peak 234

A:

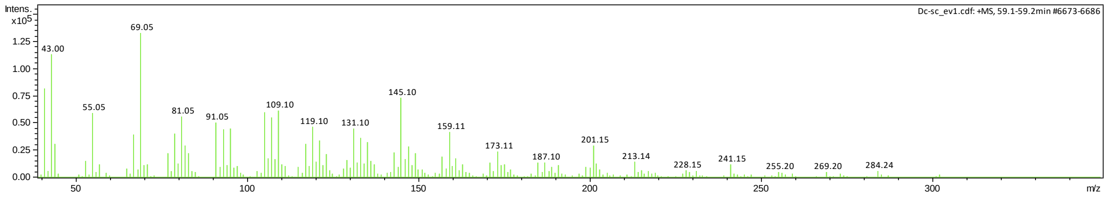

B:

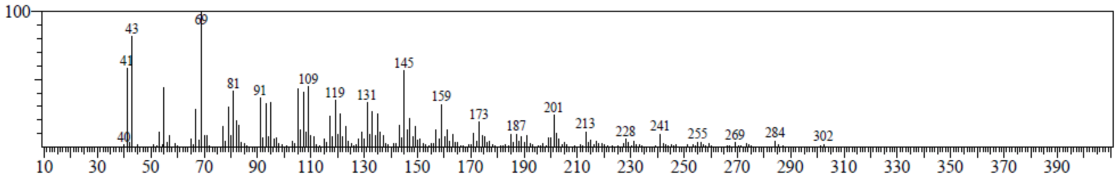

Peak 234 analyzed by gas chromatograph coupled to a mass spectrometer (GCMS-QP2010 Plus, Shimadzu, Japan) with HP-5MS capillary column (present study). A: deconvoluted mass spectrum; B: non-deconvoluted mass spectrum.

Supplementary Figure S11: peak 190

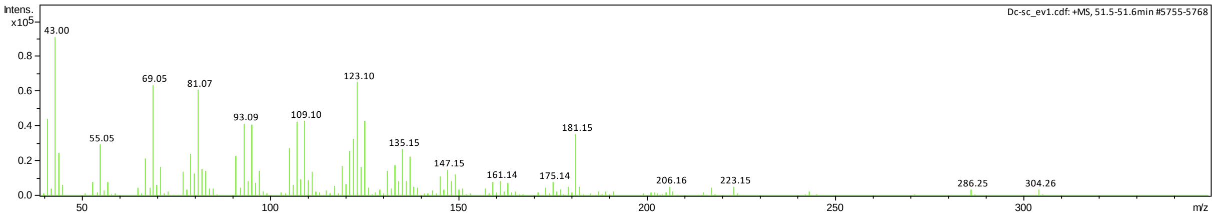

Peak 190 analyzed by gas chromatograph coupled to a mass spectrometer (GCMS-QP2010 Plus, Shimadzu, Japan) with HP-5MS capillary column (present study).

Supplementary Figure S12: peak 201

A:

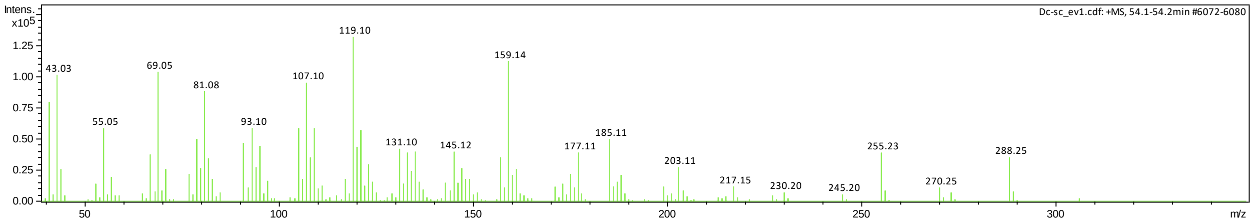

B:

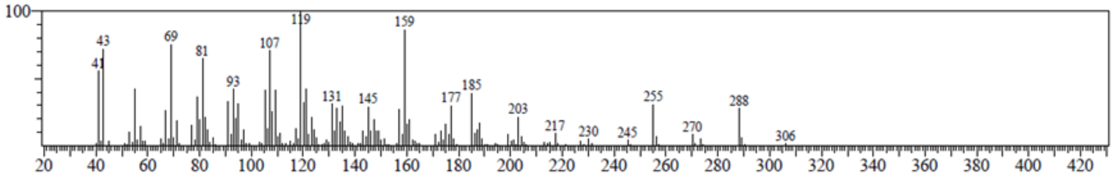

Peak 201 analyzed by gas chromatograph coupled to a mass spectrometer (GCMS-QP2010 Plus, Shimadzu, Japan) with HP-5MS capillary column (present study). A: deconvoluted mass spectrum; B: non-deconvoluted mass spectrum.

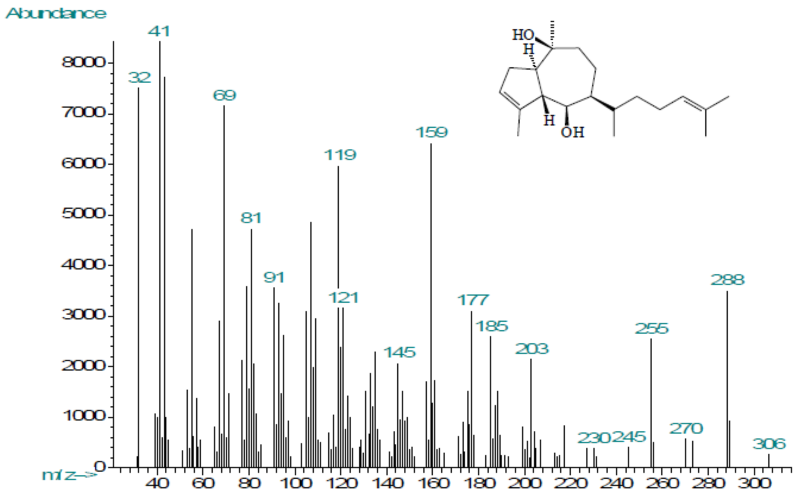

Dictyol C analysed by gas chromatograph coupled to a mass spectrometer HP6890 series GC system (Agilent) with HP 5973 mass selective detector; HP-1MS capillary column (Cavalcanti 2004).

Supplementary Figure S13: peak 193

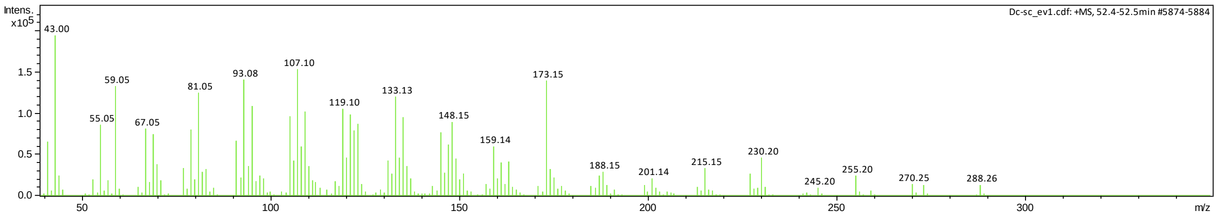

Peak 193 analyzed by gas chromatograph coupled to a mass spectrometer (GCMS-QP2010 Plus, Shimadzu, Japan) with HP-5MS capillary column (present study).

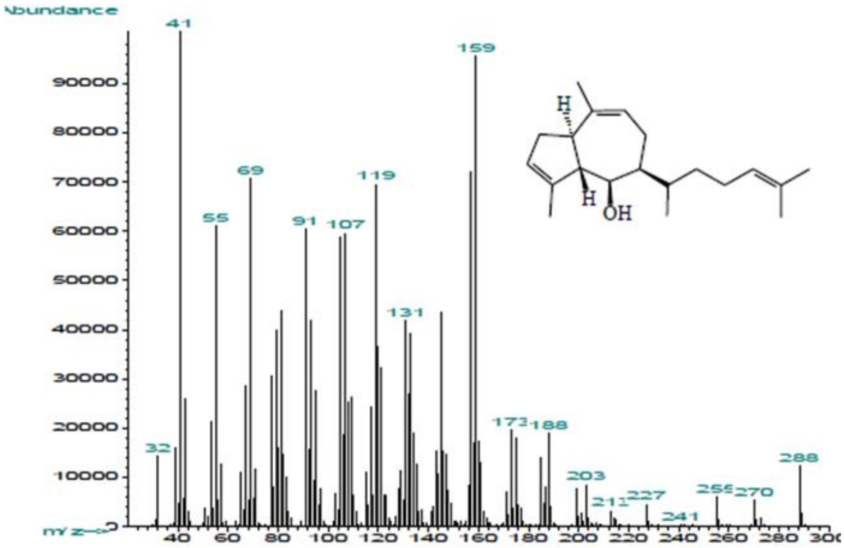

Isopachydictyol A (6) analyzed by gas chromatograph coupled to a mass spectrometer HP6890 series GC system (Agilent) with HP 5973 mass selective detector; HP-1MS capillary column (Cavalcanti 2004).

Supplementary Figure S14: peak 206

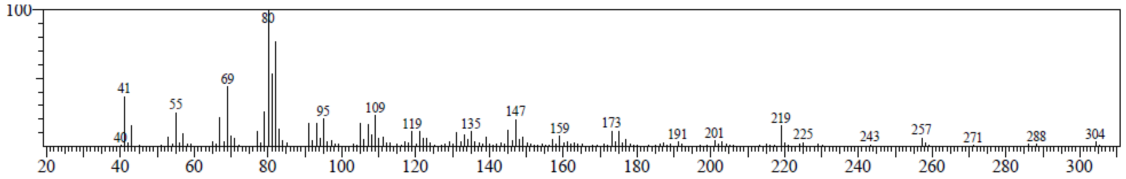

Peak 206 analyzed by gas chromatograph coupled to a mass spectrometer (GCMS-QP2010 Plus, Shimadzu, Japan) with HP-5MS capillary column (present study).

Supplementary Figure S2: peak 244

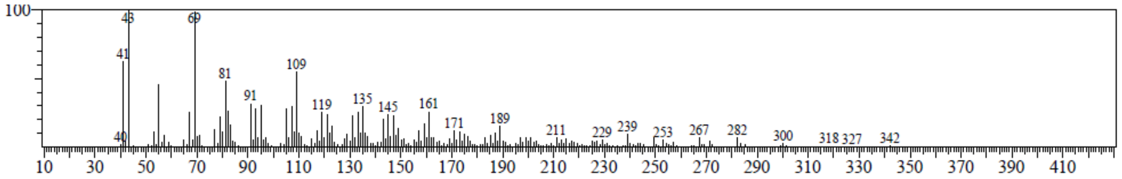

Peak 244 analyzed by gas chromatograph coupled to a mass spectrometer (GCMS-QP2010 Plus, Shimadzu, Japan) with HP-5MS capillary column (present study).

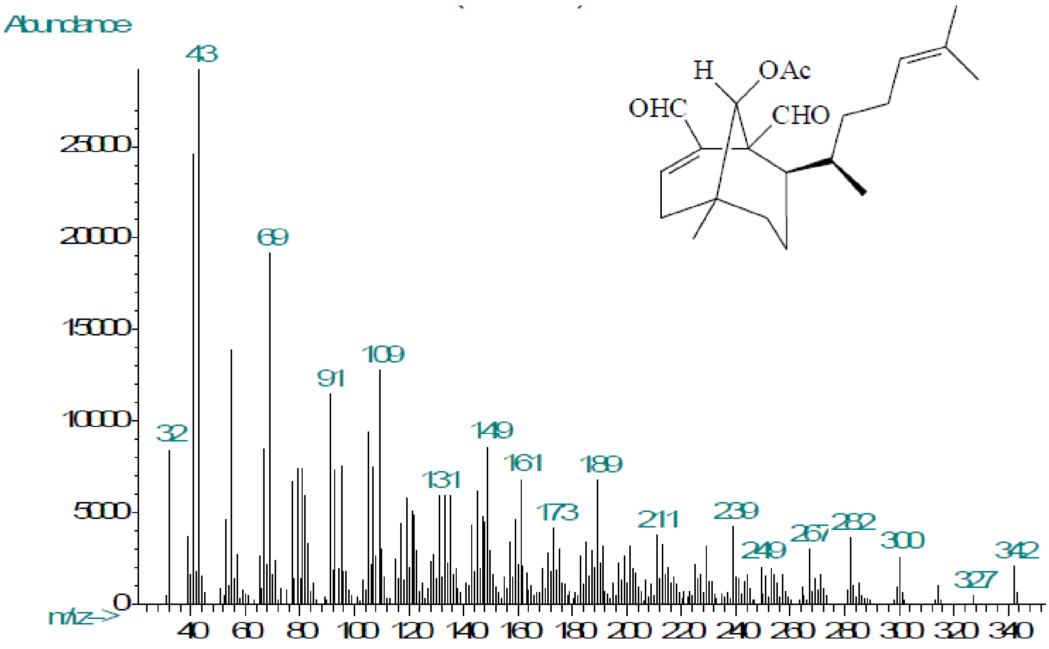

9-Acetoxydichotoma-2,13-diene-16,17-dial (8) analyzed by gas chromatograph coupled to a mass spectrometer HP6890 series GC system (Agilent) with HP 5973 mass selective detector; HP-1MS capillary column (Cavalcanti 2004).

Supplementary Figure S 3: peak 251

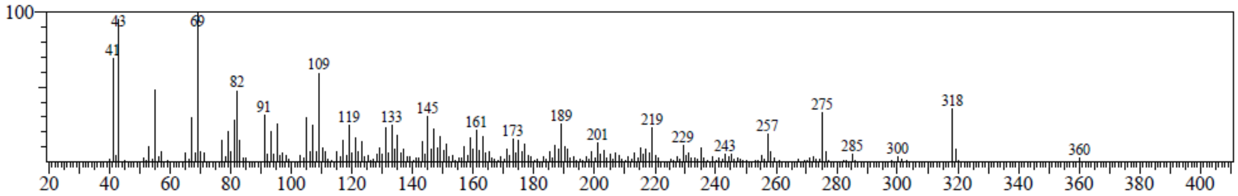

Peak 251 analyzed by gas chromatograph coupled to a mass spectrometer (GCMS-QP2010 Plus, Shimadzu, Japan) with HP-5MS capillary column (present study).

***Laurencia* and *Laurenciella* set**

The main fragments of peak 24 are *m/z* 98 (100%, rel. int) and *m/z* 135 (38) (Supplementary Figure S). When compared with literature, the authors found as main fragments for 7-epi-silphiperfolan-6β-ol *m/z* 98 (100) and *m/z* 135 (75) (Wright et al. 1990); *m/z* 98 (100) and *m/z* 135 (98%) (Weyerstahl et al. 1998). Based on general mass spectra we can suggest peak 24 belonging to a triquinane alcohol class (Supplementary Figure S1

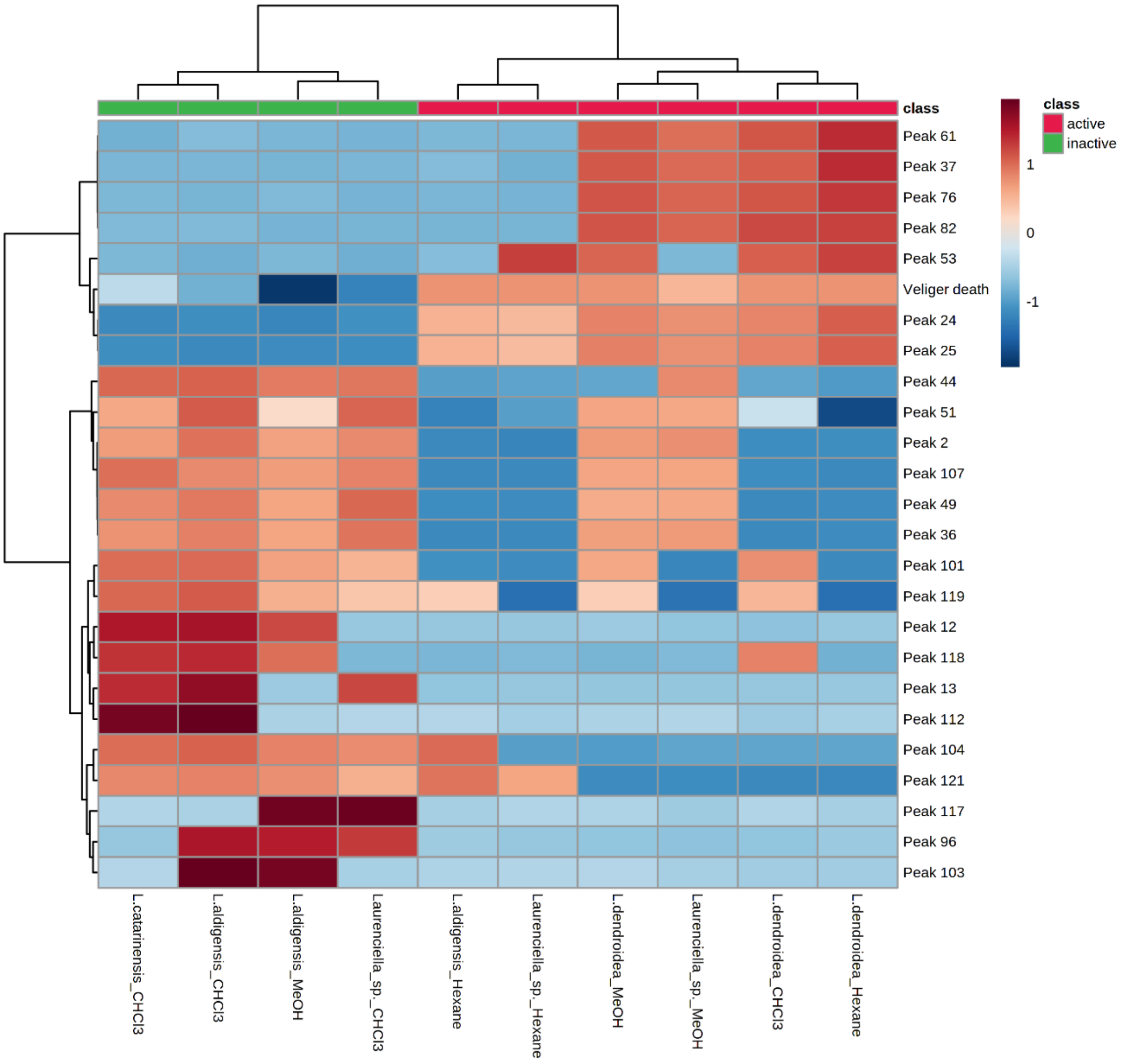

Heatmap of the top 25 features from the 123 compounds detected in the GC-MS analysis of the extracts of *Laurencia aldingensis*, *Laurencia catarinensis*, *Laurencia dendroidea*, *Laurenciella* sp. and the molluscicidal activity against *Biomphalaria glabrata* veliger embryos.

Supplementary Figure S2

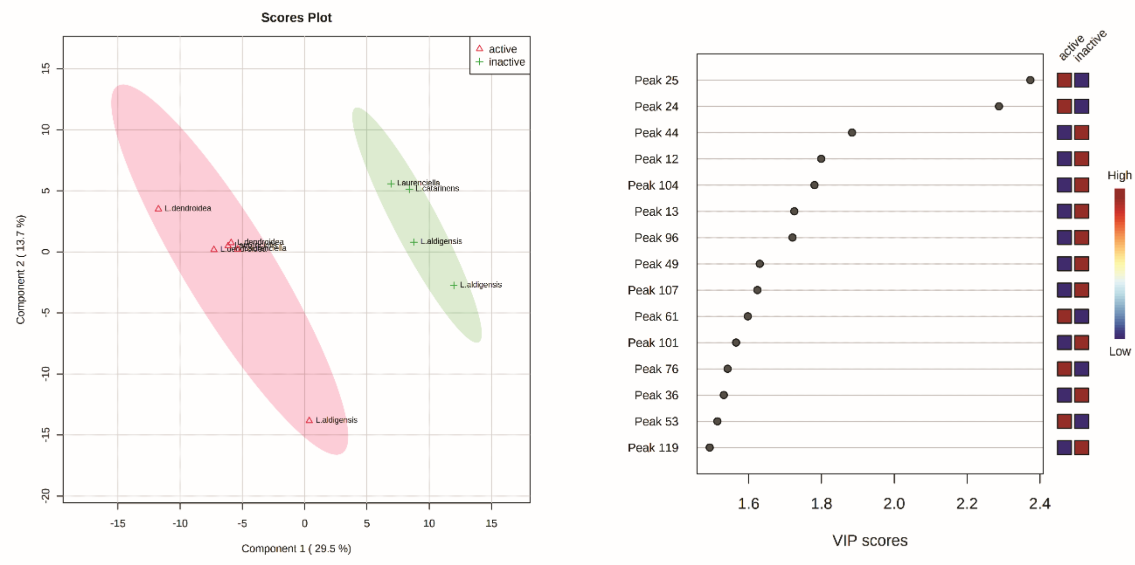

PLS-DA analysis considering the 123 compounds detected in the GC-MS analysis of the extracts of *Laurencia aldingensis*, *Laurencia catarinensis*, *Laurencia dendroidea*, *Laurenciella* sp. and the molluscicidal activity against *Biomphalaria glabrata* veliger embryos.

Supplementary Figure S3

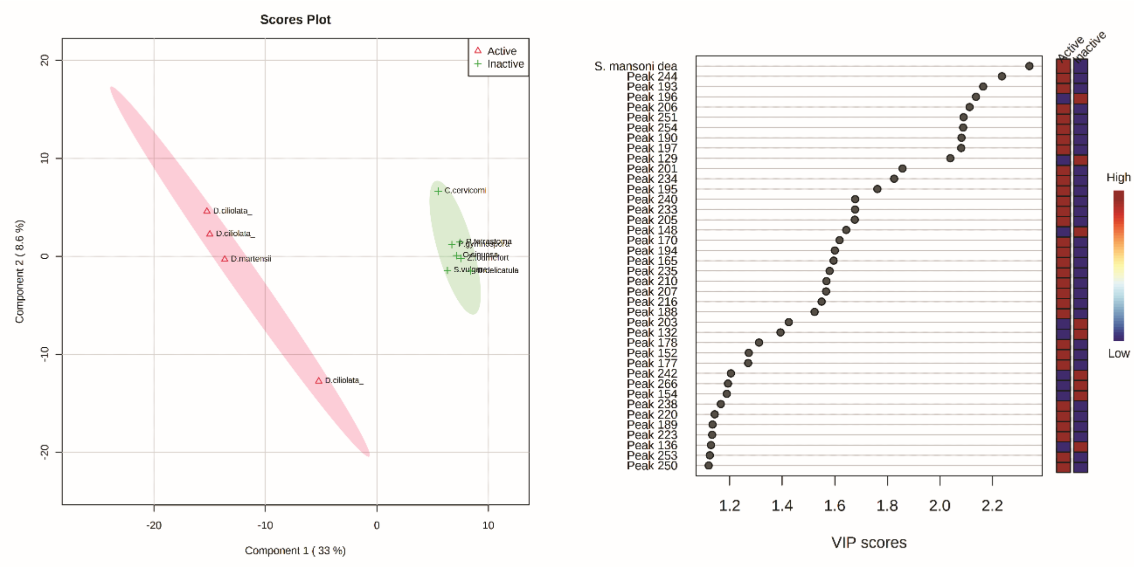

PLS-DA analysis considering the 136 compounds detected in the GC-MS analysis of the extracts of Ochrophyta and the anthelmintic activity against *Schistosoma mansoni* blastula embryos.

Supplementary Figure S4

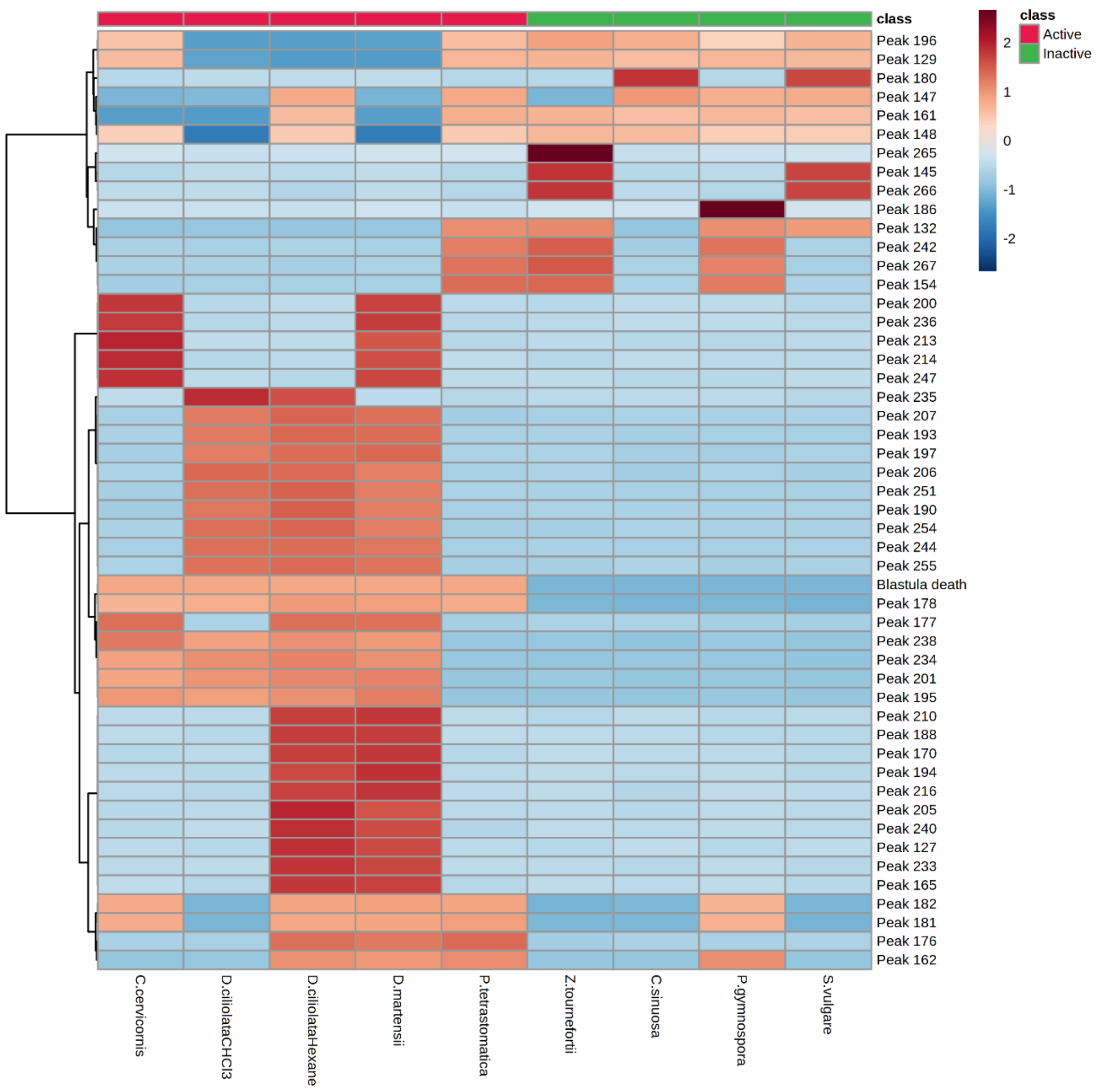

Heatmap of the top 50 features from the 136 compounds detected in the GC-MS analysis of the extracts of Ochrophyta and the molluscicidal activity against *Biomphalaria glabrata* blastula embryos.

Supplementary Figure S5

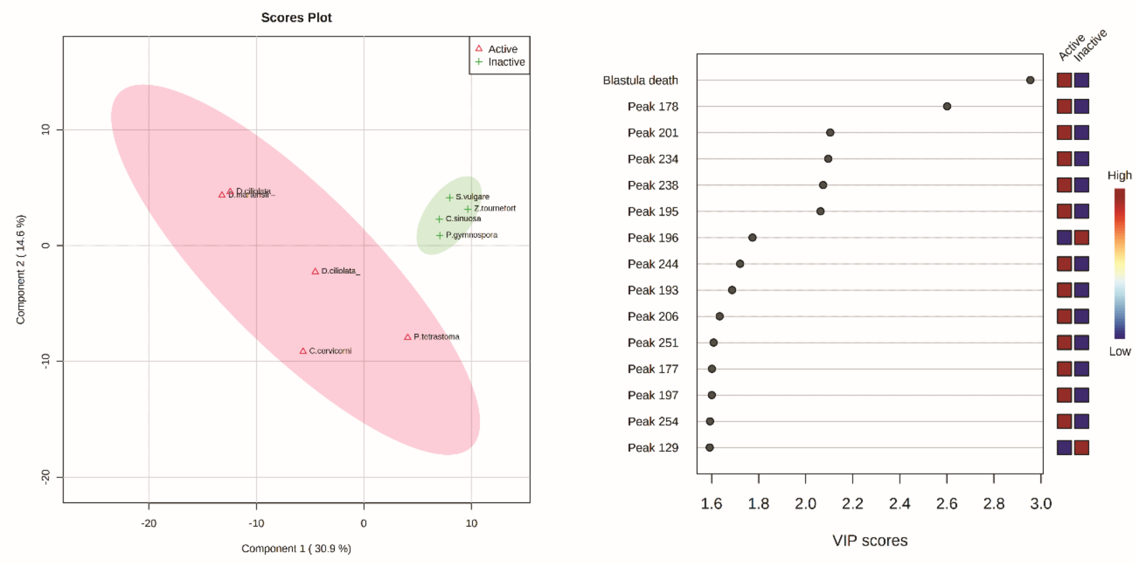

PLS-DA analysis considering the 136 compounds detected in the GC-MS analysis of the extracts of Ochrophyta and the molluscicidal activity against *Biomphalaria glabrata* blastula embryos.

Supplementary Figure S-A).

The main fragments of peak 25, *m/z* 86 (100), 81 (41), 95 (29) and 135 (22) and the mass spectrum, corroborates with literature (Supplementary Figure S). Coll & Wright (1989a) found *m/z* 86 (100), 81 (35), 135 (28) and 95 (21) and Gressler (2011) got *m/z* 86 (100), 81 (28), 135 (28), and 95 (20) for the compound silphiperfolan-7β-ol (*m/z* $[M]^+$ 222, $C_{15}H_{26}O$) isolated from *L. dendroidea*, same as present work.

***Dictyota* set**

According to studies of Brazilian populations, *D. ciliolata* and *D. mertensii* produces diterpenes from Group I (prenylated guaiane) and group III (Xenianes and Dichotomanes) (Teixeira and Kelecom 1988, 1989; Teixeira 2013).

Compounds from the group I are diterpene alcohols with a hydroazulene skeleton, e.g., dictyol A, dictyol B, dictyol C, isopachydictiol A, dictyotadiol; and from group III, e.g.

9-acetoxydichotoma-2,13-diene-16,17-dial and 4β-acetoxydictyodial (Supplementary Figure S1

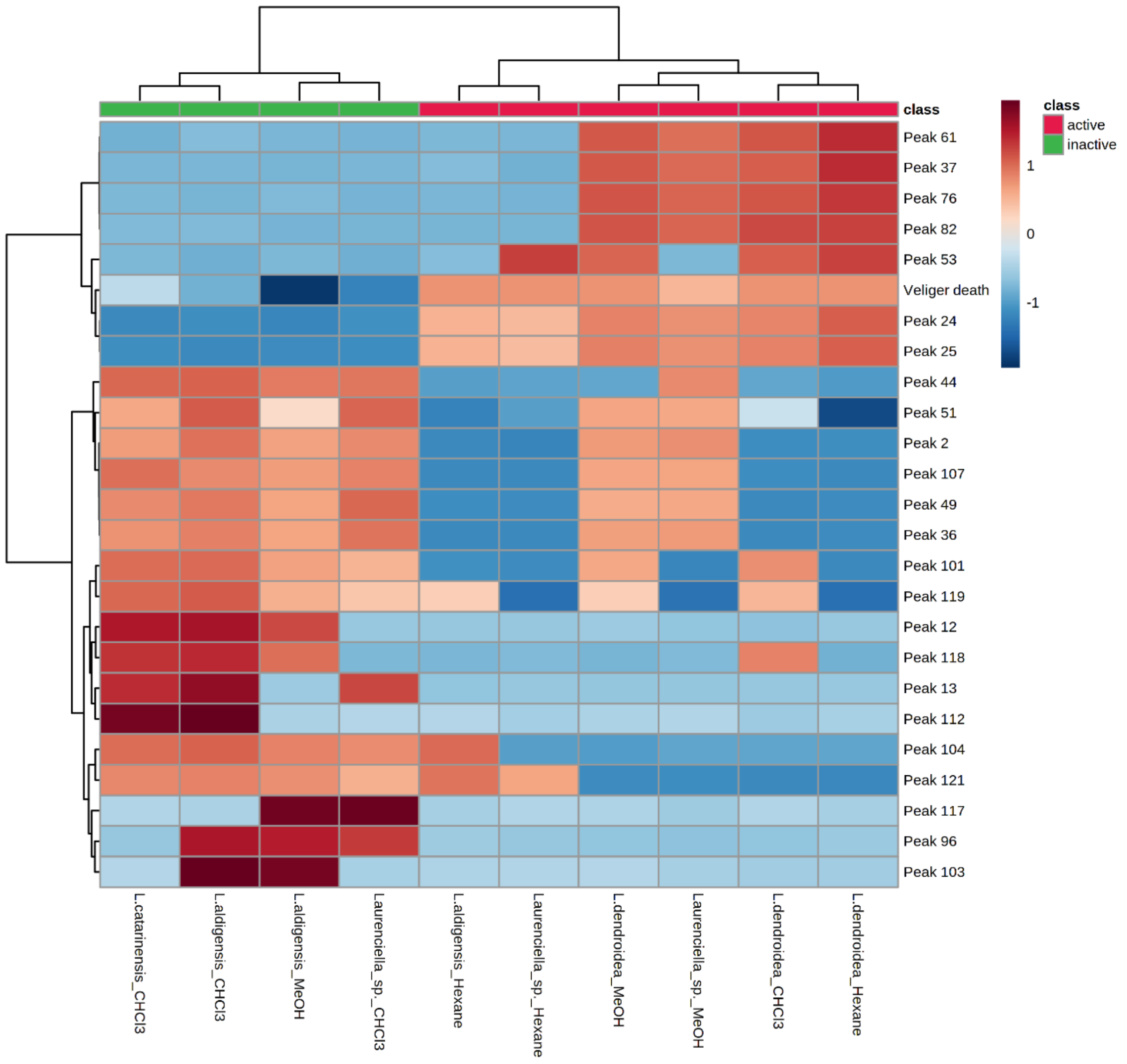

Heatmap of the top 25 features from the 123 compounds detected in the GC-MS analysis of the extracts of *Laurencia aldingensis*, *Laurencia catarinensis*, *Laurencia dendroidea*, *Laurenciella* sp. and the molluscicidal activity against *Biomphalaria glabrata* veliger embryos.

Supplementary Figure S2

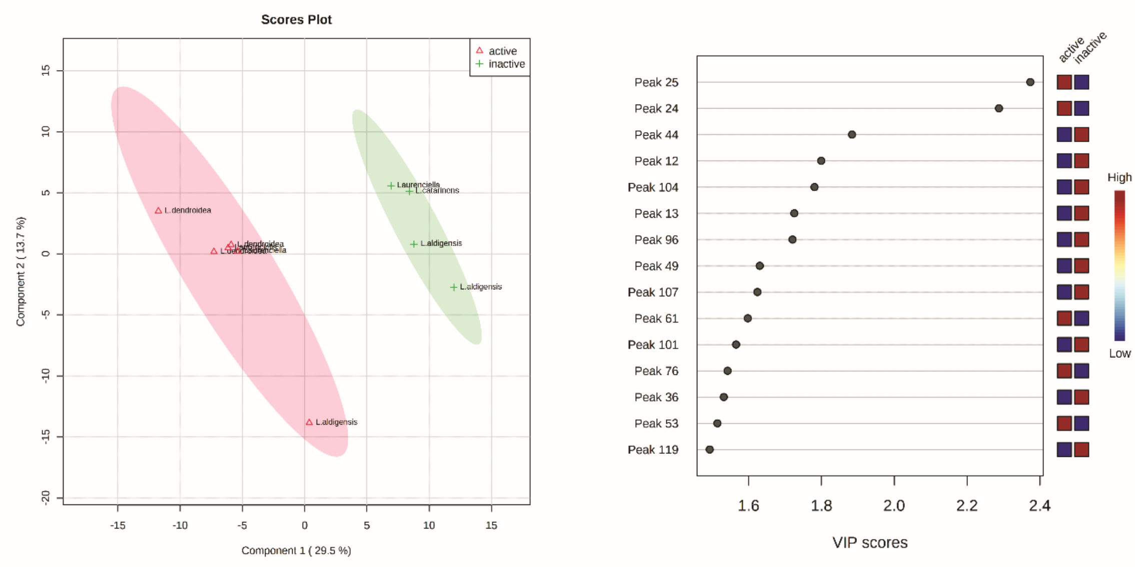

PLS-DA analysis considering the 123 compounds detected in the GC-MS analysis of the extracts of *Laurencia aldingensis*, *Laurencia catarinensis*, *Laurencia dendroidea*, *Laurenciella* sp. and the molluscicidal activity against *Biomphalaria glabrata* veliger embryos.

Supplementary Figure S3

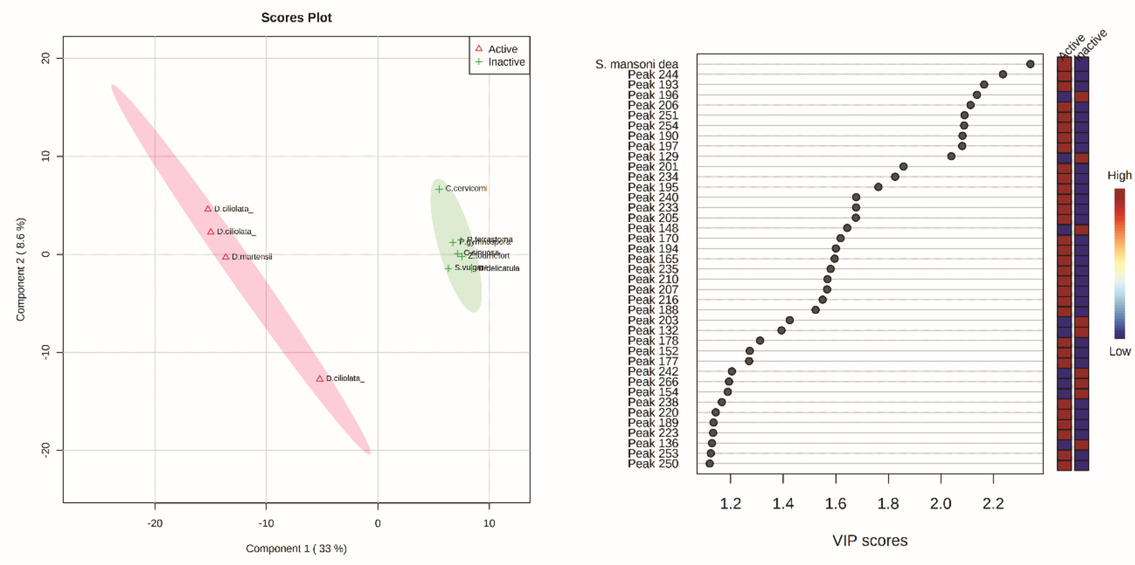

PLS-DA analysis considering the 136 compounds detected in the GC-MS analysis of the extracts of Ochrophyta and the anthelmintic activity against *Schistosoma mansoni* blastula embryos.

Supplementary Figure S4

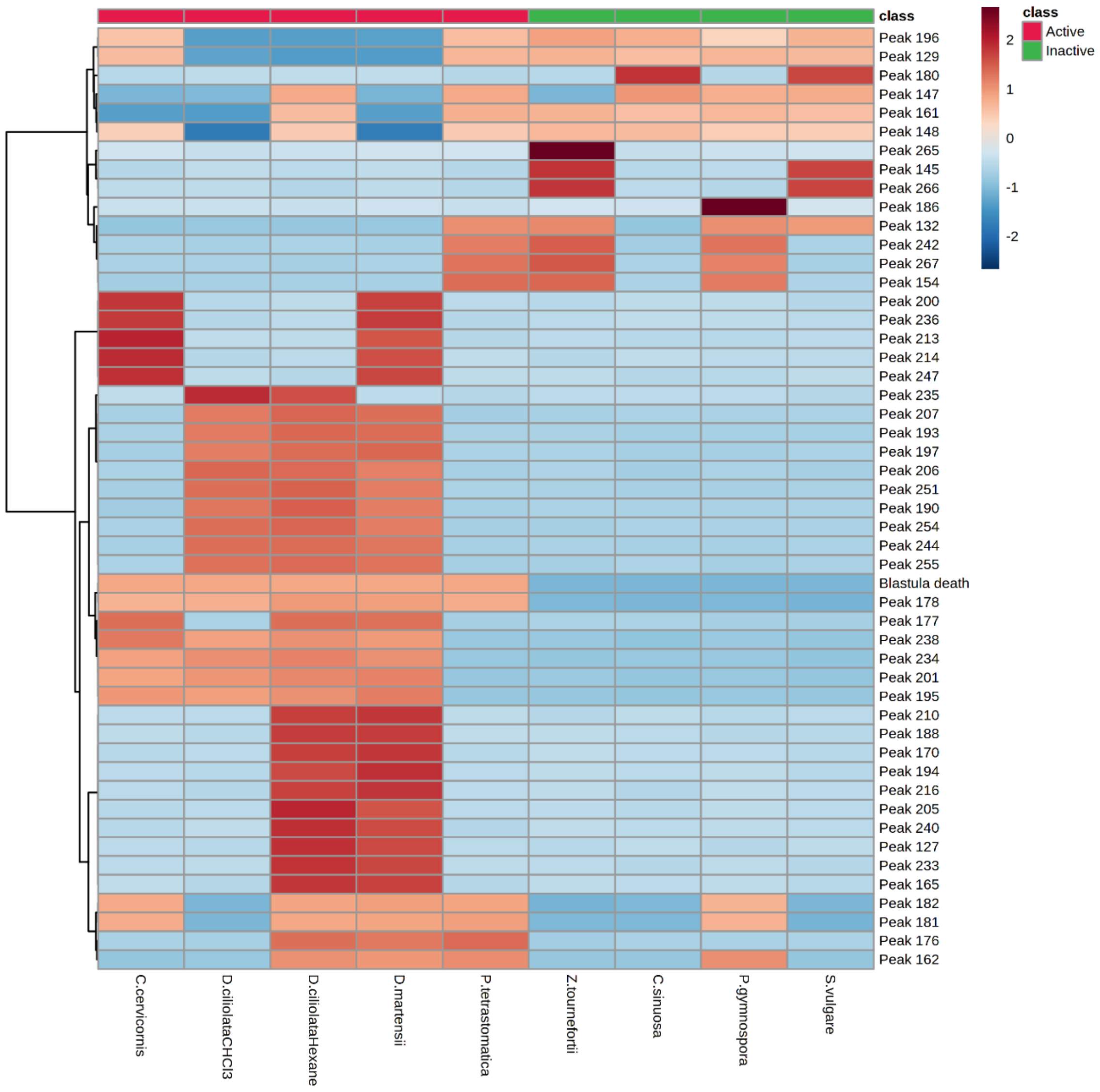

Heatmap of the top 50 features from the 136 compounds detected in the GC-MS analysis of the extracts of Ochrophyta and the molluscicidal activity against *Biomphalaria glabrata* blastula embryos.

Supplementary Figure S5

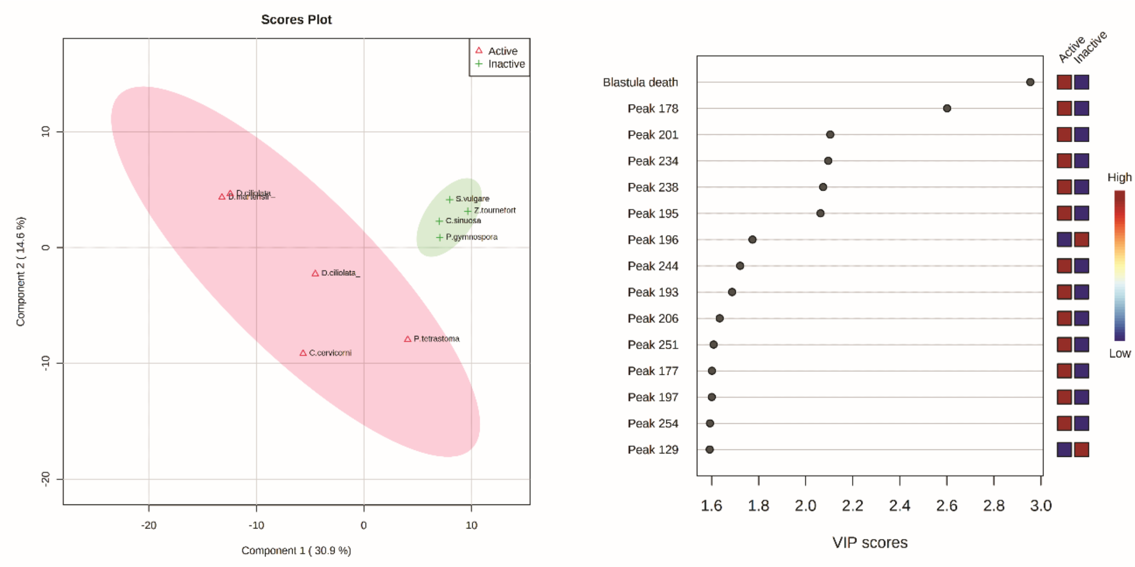

PLS-DA analysis considering the 136 compounds detected in the GC-MS analysis of the extracts of Ochrophyta and the molluscicidal activity against *Biomphalaria glabrata* blastula embryos.

Supplementary Figure S-B).

In mass spectra of the diterpenes dictyol A, dictyol C and isopachydictyol A the authors observed the presence of the fragment *m/z* 255, usual in terpenic alcohols, and the fragments *m/z* 109 $(C_8H_{13})^+$ and m/z 69 $(C_5H_9)^+$ indicating the presence of noncyclic side chain in the structure of the prenylated guaianes, dichotomane and xeniane.

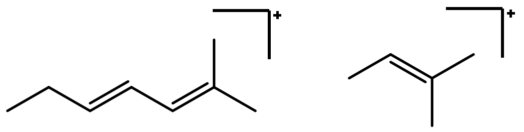

m/z 109 *m/z* 69

Fragments *m/z* 109 $(C_8H_{13})^+$ and *m/z* 69 $(C_5H_9)^+$

The fragment *m/z* 159 indicated the presence of bicyclic ring, very common in *Dictyota* species compounds like prenylated guaianes dictyol C [M-2H_2O-side chain]$^+$, dictyoxide,

isopachydictyol A [M-H_2O-side chain]$^+$, dictyotadiol. Furthermore, the consecutive losses of 2 molecules of water in the mass spectrum suggest that both oxygen atoms are present in hydroxyl groups, as we can see in dictyol C (Danise et al. 1977).

Based on these usual fragments for those compounds, we could detect them in the peaks 193, 201, 206, 254, and 234.

In the peak 234, our mass spectrum important fragments are seen at *m/z* 302 [M]$^+$ (2%, $C_{20}H_{30}O_2$); 284 [M-H_2O]$^+$ (4.4, $C_{20}H_{28}O$); 273 (2.3); 269 [284-Me]$^+$ (3.4, $C_{19}H_{25}O$); 255 (3.6); 241 [284-*i*-Pr]$^+$ (9.2, $C_{17}H_{21}O$); 201 [284-C_6H_{11}(side chain)]$^+$ (22.7, $C_{14}H_{17}O$);173 [284-side chain]$^+$ (18.2, $C_{12}H_{13}O$); 159 [173-Me]$^+$ (31.3, $C_{11}H_{11}O$); herein other prominent fragments 145 (55.3); 109 (45.8); 69 (100); 43 (83.7) (Supplementary Figure S).

In peak 201 mass spectrum diagnostically important peaks are seen at *m/z* 306 [M]$^+$ (1.7%, $C_{20}H_{34}O_2$); 288 [M-H_2O]$^+$ (27, $C_{20}H_{32}O$); 270 [M-2H_2O]$^+$ (8.4, $C_{20}H_{30}$); 177 [288-side chain]$^+$ (29.9, $C_{12}H_{17}O$); 159 [270-side chain] (85.5, $C_{12}H_{15}$); 119 (100); 107 (71.1); 81 (66.3); 69 (78.2); 43 (75.7); 41 (59) (Supplementary Figure S).

For peak 193 important mass spectra detected in our data are seen at 288 [M]$^+$ (6.2%, $C_{20}H_{32}O$); 270 [M-H_2O]$^+$ (6.7, $C_{20}H_{30}$); 255 (12.4); 201 [270-C_5H_9 (side chain)]$^+$ (10.6, $C_{15}H_{21}$); 188 [201-Me]$^+$ (14.5, $C_{14}H_{20}$); 173 (71.3); 159 [270-side chain]$^+$ (30.2, $C_{12}H_{15}$); 148 (45.9); 145 [188-*i*-Pr]$^+$ (39.1, $C_{11}H_{13}$); 133 (61.5); 119 (53.9); 107 (78.6); 93 (71.9); 81 (63.8); 67 (41.8); 59 (67.6); 55 (43.8); 43 (100); 41 (33.6) (Supplementary Figure S).

Peak 206 main mass peaks are at 304 [M]$^+$ (3.4%); 288 (1.8); 286 [M-H2O]$^+$ (1.9, $C_{20}H_{30}O$); 271 [286-Me]$^+$ (0.3, $C_{29}H_{27}O$); 257 (6.8); 243 (0.3); 225 (3.0); 219 (15); 201 [271-C_5H_9]$^+$ (5.7, $C_{14}H_{17}O$); 173 (13.6); 159 [201- C_3H_5]$^+$ (12.1, $C_{11}H_{11}O$); 147 (24.4); 135 (14.6); 131 (15.5); 119 (18.6); 109 (30.6); 95 (27.6); 80 (100); 69 (57.6, C_5H_9); 55 (33.9); 41 (50.8, C_3H_5) (Supplementary Figure S).

The presence of the fragment 69 also indicates the loss of part of side chain in the peak 190. The main fragments are seen at 304 [M]$^+$ (3.8%); 286 [M-H_2O]$^+$ (3.2, $C_{2O}H_{30}O$); 261 [M-*i*-Pr]$^+$ (0.6, $C_{17}H_{25}O_2$); 243 [261-H_2O]$^+$ (2.4, $C_{17}H_{23}O$); 215 (1.7); 175 [286-side chain]$^+$ (8, $C_{12}H_{15}O$); 157 [175- H_2O]$^+$ (4, $C_{12}H_{13}$); 142 [157-Me]$^+$ (1.3, $C_{11}H_{10}$); 129

[159-2Me] (3.2, $C_{10}H_9$); 95 (44.6); 93 (45.2); 91 (24.5); 81 (68.0); 79 (25.9); 69 (68.9); 43 (100); 41 (46.6).

Freitas et al. (2007) studied *D. mertensii* (same specie as present study), and described the main fragments brought forth by HRCG-MS of dictyol B which we will mention some: 304 (1); 286 (50); 215 (9); 157 (100); 142 (27); 129 (29); 91 (35); 79 (27); 69 (45); 55 (29); 41 (45). Our peak 190 is probably an analogue of dictyol B (Supplementary Figure S).

Fatorusso et al. (1976) isolated from *D. dichotoma* the two diterpenes alcohols dictyol A and B, and they described for dictyol A the presence of fragments at *m/z* 284, 273, 269, and 255. The selected peak 234 shows the fragments *m/z* 284, 269, and 255 but not the *m/z* 237 (Supplementary Figure S).

References

Ayyad, S.E.; Makki, M.S.; Al-Kayal, N.S.; Basaif, S.A.; El-Foty, K.O.; Asiri, A.M.; Alarif,W.M.; Badria, F.A. Cytotoxic and protective DNA damage of three new diterpenoids from the brown alga *Dictoyota dichotoma*. Eur. J. Med. Chem. 2011, 46, 175–182.

Cavalcanti DN, Rezende CM, Pinto AC, Teixeira VL 2006. Diterpenoid constituents from the brown alga *Dictyota menstrualis* (Dictyotaceae, Phaeophyta). Nat Prod Comm 1: 609-611

Cavalcanti, D N. Caracterização química da alga parda *Dictyota menstrualis* (Dictyotales, Phaeophyta) e atividade antiviral de seus diterpenos. Tese de doutorado - UFRJ/IQ Rio de Janeiro, 229 p, 2004.

Danise, B., Minale, L., Riccio R., Amico V., Oriente G., Piattelli M., Tringali C., Fattorusso E., Magno S. and Mayol L. 1977. Further perhydroazulene diterpenes from marine organisms. Experientia 33, 413–415

De-Paula JC, Bueno LB, Cavalcanti DN, Yoneshigue-Valentin Y, Teixeira VL. Diterpenes from the Brown Alga *Dictyota crenulata*. Molecules. 2008;13(6):1253-1262.

De-Paula, JC; Lopes-Filho, EAP; Salgueiro, F; Yoneshigue-Valentin, Y; Cavalcanti, DN; Villaça, RC & Teixeira VL (2018). Diterpenes content of the brown alga *Dictyota ciliolata* (Dictyotales, Phaeophyceae) and recognition of a Brazilian haplotype based on psbA sequences, New Zealand Journal of Botany, 56:4, 415-429, DOI: 10.1080/0028825X.2018.1535441.

Ernesto Fattorussos E, Magno S; Mayol L; Santacroce C; Sica D. 1976. Dictyol A and B, two novel diterpene alcohols from the brown alga *Dictyota Dichotoma.* J.C.S. Chem. Comm.

Freitas O do SP, de Oliveira AS, De-Paula JC, Pereira RC, Cavalcanti DN, Teixeira VL. 2007. Chemical Variation in the Diterpenes from the Brazilian Brown Alga *Dictyota Mertensii* (Dictyotaceae, Phaeophyta). Natural Product Communications 2: 13-15. doi:10.1177/1934578X0700200104

Gressler V, Stein EM, Dörr F, Fujii MT, Colepicolo P, Pinto E 2011. Sesquiterpenes from the essential oil of *Laurencia dendroidea* (Ceramiales, Rhodophyta): isolation, biological activities and distribution among seaweeds. Rev Bras Farmacogn 21: 248-254.

Ortiz-Ramírez, FA; Cavalcanti, DN; Villaça, RC; De-Paula, JC; Yoneshigue-Valentin, Y; Teixeira VL. 2008. Chemical Variation in the Diterpenes from the Brazilian Brown Alga *Dictyota menstrualis* (Dictyotaceae, Phaeophyceae). Natural Product Communications, 11: 1747-1940

Teixeira, V. L. 2013. Produtos Naturais de Algas Marinhas Bentônicas. Rev. Virtual Quim. 5 (3): 343-362.

Teixeira, V.L.; Kelecom, A. A chemotaxonomic study of diterpenes from marine brown algae of the genus *Dictyota*. Sci. Total Environ. 1988, 75, 271–283.

Weyerstahl P, Marschall H, Seelmann I, and Jakupovic J 1998. Cameroonane, Prenopsane and Nopsane, Three New Tricyclic Sesquiterpene Skeletons. Eur. J. Org. Chem. 1205-1212

Wright AD, Coll JC, Price IR 1990. Tropical marine algae, VII The chemical composition of marine algae from North Queensland waters. J Nat Prod 33: 845-861.
